# Effects of Rich in Β-Glucans Edible Mushrooms on Aging Gut Microbiota Characteristics: An In Vitro Study

**DOI:** 10.3390/molecules25122806

**Published:** 2020-06-18

**Authors:** Evdokia K. Mitsou, Georgia Saxami, Emmanuela Stamoulou, Evangelia Kerezoudi, Eirini Terzi, Georgios Koutrotsios, Georgios Bekiaris, Georgios I. Zervakis, Konstantinos C. Mountzouris, Vasiliki Pletsa, Adamantini Kyriacou

**Affiliations:** 1 Department of Nutrition and Dietetics, Harokopio University, 17671 Athens, Greece; emitsou@hua.gr (E.K.M.); gsaxami@hua.gr (G.S.); dp4421749@hua.gr (E.S.); dp4421804@hua.gr (E.K.); dp4521704@hua.gr (E.T.); 2 Laboratory of General and Agricultural Microbiology, Department of Crop Science, Agricultural University of Athens, 11855 Athens, Greece; georgioskoutrotsios@gmail.com (G.K.); giorgosbekiaris@yahoo.gr (G.B.); zervakis@aua.gr (G.I.Z.); 3 Department of Nutritional Physiology and Feeding, Agricultural University of Athens, 11855 Athens, Greece; kmountzouris@aua.gr; 4 Institute of Chemical Biology, National Hellenic Research Foundation, 11635 Athens, Greece; vpletsa@eie.gr

**Keywords:** gut microbiota, SCFAs, aging, prebiotics, β-glucans, edible mushroom, *Pleurotus ostreatus*, *Pleurotus eryngii*, *Hericium erinaceus*, *Cyclocybe cylindracea*

## Abstract

Alterations of gut microbiota are evident during the aging process. Prebiotics may restore the gut microbial balance, with β-glucans emerging as prebiotic candidates. This study aimed to investigate the impact of edible mushrooms rich in β-glucans on the gut microbiota composition and metabolites by using in vitro static batch culture fermentations and fecal inocula from elderly donors (n = 8). *Pleurotus ostreatus*, *P. eryngii*, *Hericium erinaceus* and *Cyclocybe cylindracea* mushrooms derived from various substrates were examined. Gut microbiota composition (quantitative PCR (qPCR)) and short-chain fatty acids (SCFAs; gas chromatography (GC)) were determined during the 24-h fermentation. *P. eryngii* induced a strong lactogenic effect, while *P. ostreatus* and *C. cylindracea* induced a significant bifidogenic effect (*p* for all <0.05). Furthermore, *P. eryngii* produced on wheat straw and the prebiotic inulin had comparable Prebiotic Indexes, while *P. eryngii* produced on wheat straw/grape marc significantly increased the levels of tested butyrate producers. *P. ostreatus*, *P. eryngii* and *C. cylindracea* had similar trends in SCFA profile; *H. erinaceus* mushrooms were more diverse, especially in the production of propionate, butyrate and branched SCFAs. In conclusion, mushrooms rich in β-glucans may exert beneficial in vitro effects in gut microbiota and/or SCFAs production in elderly subjects.

## 1. Introduction

Gut microbiota, one of the most complex and dense microbial ecosystems, is recognized for its crucial role on human health through a variety of functions, such as the extraction of energy from foods, alterations in the appetite signaling pathway [1,2], involvement in host metabolic processes [3,4], host protection against pathogenic microorganisms [5,6] and immune system development and function [7]. Imbalance in the dynamic interactions among microbial intestinal populations, a phenomenon called dysbiosis, has been related to many pathological conditions, e.g., inflammatory bowel disease, irritable bowel syndrome, obesity, colorectal cancer [8,9]. The restoration of the normal intestinal microbiota can be acquired through the consumption of dietary factors, such as probiotics, prebiotics or synbiotics [10]. Prebiotics are defined as ‘nondigestible food ingredients that when administered, exert a beneficial effect on the host health’ [11]. They advance the growth of lactic acid bacteria and bifidobacteria in the colon, while exerting antagonistic action on harmful microorganisms [11]. The most recognized prebiotics are fructo-oligosaccharides (FOS), galacto-oligosaccharides (GOS), lactulose and inulin [12].

The beneficial effects of prebiotics have fueled the research for novel and alternative sources. Among the emerging prebiotic candidates are β-glucans, i.e., polysaccharides receiving increasing attention due to their human health benefits [13,14]. B-glucans are abundant in the bran of certain cereal grains (oat and barley), and also in various mushroom species [15,16]. Fungal β-glucans are beta-(1→3,1→6)-D-glucans, whereas their cereal-derived counterparts are beta-(1→3/1→4)-D-glucans [17].

Fermentation of prebiotics by intestinal microbial consortia results in the increased production of short-chain fatty acids (SCFAs), with key roles in the prebiotic function [18]. Acetate, propionate and butyrate are the most abundant (≥95%) SCFAs in the human colon and stool. These microbial metabolites have been associated with many beneficial effects on host health, such as the inhibition of pH-sensitive pathogens, the increase of mineral absorption, the regulation of intestinal motility and the enhancement of the intestinal epithelial barrier [18,19]. Acetate is transported from the gut by the portal vein in the liver, where it participates in lipogenesis, and also in distant body sites, and it can be metabolized in the human muscle, kidney, heart and brain [20]. Propionate exerts anti-lipogenic, cholesterol-lowering, anti-inflammatory and anti-carcinogenic activities [21]. Butyrate is the main energy source for intestinal epithelial cells, while it also modulates the cell growth and differentiation of intestinal cells, exerts strong anti-inflammatory activity, stimulates the cell apoptosis and exhibits strong anticancer properties [22,23,24].

In vitro intestinal fermentation models have been developed as a powerful tool for examining the impact of prebiotic substrates on the composition and metabolic activity of gut microbial populations [25]. There are several studies investigating the prebiotic potential using fecal inoculum from infants or healthy adults, but there are only a few relevant data for elderly populations [26]. In addition, only a few in vitro fermentation models have been developed in order to evaluate the effects of edible mushrooms (i.e., lyophilized powder from the entire fruitbody) [27], whereas several exist about the study of mushrooms extracts [28,29,30], or mushrooms β-glucans [31,32], on the composition and/or activity of the gut microbiota.

The aim of this work was to investigate the impact of rich in β-glucans edible mushrooms (derived from six strains of *Cyclocybe cylindracea*, *Hericium erinaceus*, *Pleurotus eryngii* and *P. ostreatus* isolated from Greek habitats and cultivated in various substrates) on the gut microbiota composition and metabolites production using an in vitro batch culture fermentation system inoculated with fecal samples from apparently healthy volunteers over 65 years old.

## 2. Results

Fungal strains and mushroom cultivation substrates appear in Table 1, together with the mushrooms content in total, α- and β-glucans. On the basis of the results presented, the mushrooms were rich in β-glucans, with a mean content of 31.58% (*w/w* d.w.) and a range from 15.4% in the case of HEBS up to 42.2% (*w/w* d.w.) in the case of PEWSGM. For the in vitro static batch culture fermentation, lyophilized mushroom powder was added to the basal medium, while the prebiotic inulin (INU2) was used as positive control, (a negative control (NC) was also included; i.e., a basal medium with no carbohydrate source). This procedure was repeated eight times, using fresh fecal inoculum from apparently healthy volunteers over 65 years old. Descriptive characteristics of the fecal donors are available in Table 2. All donors were compliant with the inclusion criteria of the study, concerning health status, dietary habits or the consumption of probiotics/prebiotics/antibiotics. Drug-treated hypertension was the most frequent medical condition among donors. No allergies or recent consumption of nonsteroidal anti-inflammatory drugs was reported among volunteers. Furthermore, most subjects were normal-weighted (62.5%), moderate active (75.0%), with low daily fiber intake (75% of cases below recommended intake of 25–30 g·d^−1^).

Faecal inocula were categorized as Bristol Stool Scale (BBS) type 5 in 4 cases, with the rest being assigned in type 3 (n = 1) and 4 (n = 3); no volunteer reported diarrheic evacuations for a 7-d period before sampling day, whereas chronic constipation or gastrointestinal problems were part of exclusion criteria.

### 2.1. Gut Microbiota Analysis

At the baseline (t = 0 h), levels of total bacteria, butyrate producers and *C. perfringens* had significant differences compared to NC and/or INU2, especially in the case of PEWS and CC2WS. Nevertheless, similar initial levels of bifidobacteria, lactobacilli and *Bacteroides* spp. were detected in all mushrooms, compared to negative and positive controls (Table 3).

After 24-h fermentation, total bacterial levels significantly increased in nearly all substrates compared to NC (Table 3). Positive control (INU2) induced both a significant bifidogenic and lactogenic effect, compared to NC and baseline (t = 0 h). Nevertheless, mushrooms had a more versatile role, with *P. eryngii* inducing a strong lactogenic effect whereas representatives of *P. ostreatus* and *C. cylindracea* demonstrated a significant bifidogenic effect. Indeed, these effects were significant compared to NC and quite analogous to that of the prebiotic inulin after 24-h fermentation. Furthermore, paired-samples analysis revealed that CC2WS induced significant increase in both baseline lactobacilli and bifidobacterial levels, whereas initial bifidobacterial levels were also elevated after the fermentation with mushrooms from the POWS, POLWS, PEWS, PEWSGM and HEOLRP treatments.

*Bacteroides* spp. levels were reduced in the case of *C. cylindracea* mushrooms after 24-h fermentation, likewise NC. On the contrary, the effect of all other tested mushroom was similar to the effect of inulin on *Bacteroides* population, with stable or rather increased levels after 24-h fermentation. Clostridial levels increased in the case of PEWS, POOLRP and CC2WS, compared to baseline, whereas only POLWS had significant higher clostridial levels compared to NC after 24-h fermentation.

Butyrate producers (e.g., *Faecalibacterium prausnitzii*, *Roseburia* spp.-*Eubacterium rectale*) were significantly reduced under no carbohydrate source (NC), whereas, in the case of inulin (INU2), the levels of *F. prausnitzii* increased and levels of *Roseburia* spp.-*E. rectale* decreased. Based on our experimental data, 24-h fermentation of PEWSGM significantly increased the levels of both bacterial groups, while mushrooms deriving from several substrates had positive effects in *F. prausnitzii* population (e.g., POWS, POOLRP, POLWS, PEWS, CC505WS). Finally, in the case of HEOLRP, the levels of both butyrate producers were increased compared to NC after 24-h fermentation, though they remained rather stable compared to baseline data (t = 0 h).

Based on data from Table 3, we observed that *P. ostreatus* mushrooms cultivated in wheat straw (POWS, POLWS) were characterized by an increase in levels of bifidobacteria and *F. prausnitzii*. Furthermore, fermentation of *P. eryngii* and *C. cylindracea* mushrooms induced diverse effects in microbial profiling, irrespectively of strain (CC2WS, CC505WS) or cultivation substrate (PEWS, PEWSGM) used, whereas limited effects on the tested microorganisms were observed in the case of *H. erinaceum* treatments. In addition, the comparison of the same strain in different substrates (POWS vs. POOLRP, POLWS vs. POLTPOMW, PEWS vs. PEWSGM, HEBS vs. HEOLRP) or of different strains cultivated in the same substrate (POWS vs. POLWS, CC2WS vs. CC505WS) revealed only a significant 0.4-log mean difference in *Roseburia* sp.*-E. rectale* levels after 24-h fermentation of PEWS, compared to PEWSGM (8.07 ± 0.28 vs. 8.43 ± 0.26 log_10_ 16S copies, respectively, *p* = 0.021).

### 2.2. Prebiotic Indexes

**Figure 1:**
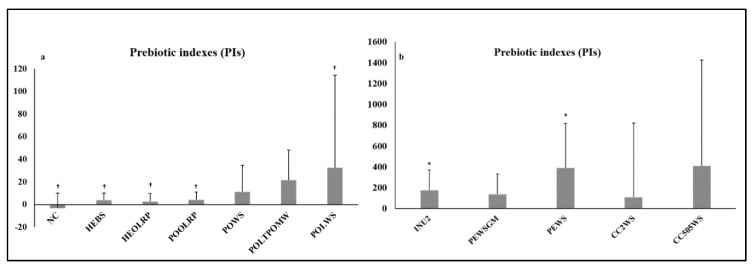
Prebiotic Indexes (PIs) of the tested mushrooms and controls after 24-h fermentation (**a**: NC, HEBS, HEOLRP, POOLRP, POWS, POLTPOMW and POLWS treatments; **b**: INU2, PEWSGM, PEWS, CC2WS and CC505WS treatments);Values are expressed as mean and SD (for POLTPOMW and HEBS n = 4—for CC2WS and CC505 n = 7); *: significant difference compared to NC (*p* < 0.05) (Mann-Whitney test); †: significant difference compared to INU2 (*p* < 0.05) (Mann-Whitney test).

Inulin and tested mushrooms demonstrated positive mean PIs after 24-h fermentation, with higher levels detected in the case of the prebiotic inulin and *P. eryngii* (PEWS, PEWSGM) and *C. cylindracea* (CC2WS, CC505WS) mushrooms (overall *p* = 0.081). Furthermore, only INU2 (*p* = 0.005) and PEWS (*p* = 0.021) resulted in significantly higher PIs compared to NC after 24-h fermentation (Figure 1a,b). It is worth mentioning that there was no difference between INU2 and PEWS (*p* = 0.401). In addition, only inulin and PEWS induced positive PIs results in all eight runs of the experiment with different fecal donors (Supplementary Materials, Table S1).

Though no significant overall differences were observed among PIs for the first four runs of the experiment with all substrates available (*p* = 0.717), once again INU2 (*p* = 0.149), PEWS (*p* = 0.149) and CC2WS (*p* = 0.149) resulted in considerably higher PIs compared to NC (Supplementary Materials, Figure S1a,b). Further analysis in mean PIs values of mushrooms indicated also no significant strain- or substrate-specific differentiation [e.g., POWS vs. POOLRP, *p* = 1.000; POLWS vs. POLTPOMW, *p* = 0.062 (*p* = 1.000 for n = 4); POWS vs. POLWS, *p* = 0.753; PEWS vs. PEWSGM, *p* = 0.674; HEBS vs. HEOLRP, *p* = 0.089 (*p* = 0.683 for n = 4); CC2WS vs. CC505WS, *p* = 0.406]. Finally, it was noticed that prebiotic potential exhibited quite high variability among the eight different fecal donors (Supplementary Materials, Table S1).

Statistical analysis revealed significant positive correlations of mean PIs values of mushrooms with their average total glucan (Spearman’s rho 0.806, *p* = 0.005) and β-glucan content (Spearman’s rho 0.758, *p* = 0.011) for all the available data and for the first four runs of the fermentation procedure (total glucan: Spearman’s rho 0.758, *p* = 0.011; β-glucan: Spearman’s rho 0.709, *p* = 0.022) (Supplementary Materials, Figure S2a–d). No significant correlations were detected in terms of α-glucan content.

### 2.3. Short Chain Fatty Acids (SCFAs) Analysis

**Figure 2:**
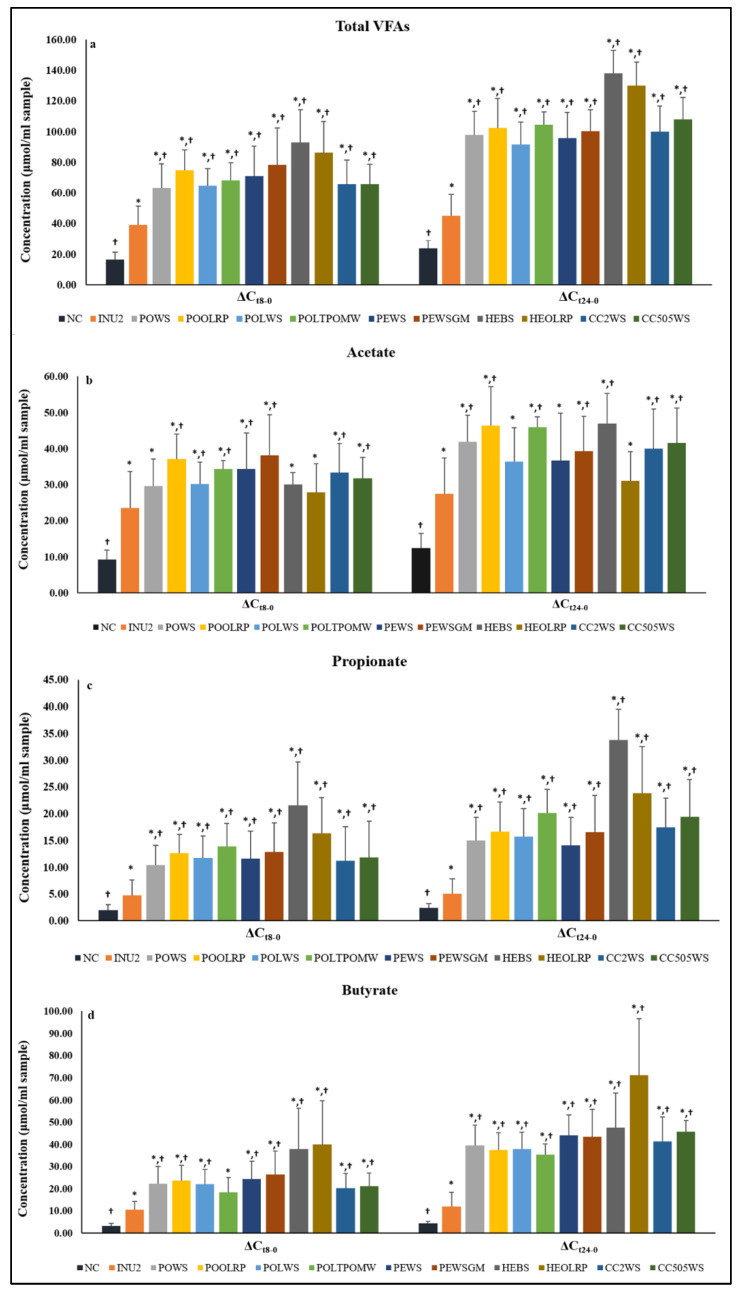
Differences (Δ) in concentrations (μmol mL^−1^) of (**a**) total volatile fatty acids (VFAs), (**b**) acetate, (**c**) propionate and (**d**) butyrate after 8-h fermentation (ΔCt8-0) and 24-h fermentation (ΔCt24-0) compared to baseline. Values are expressed as mean and SD, where ΔCt8-0 is defined as ‘Concentration t = 8 h minus Concentration t = 0 h’ and ΔCt24-0 is defined as ‘Concentration t = 24 h minus Concentration t = 0 h’ (for POLTPOMW and HEBS n = 4—for CC2WS and CC505 n = 7); *: significant difference compared to NC (*p* < 0.05) (Mann-Whitney test or *t-test*); †: significant difference compared to INU2 (*p* < 0.05) (Mann-Whitney test or *t*-test).

**Figure 3:**
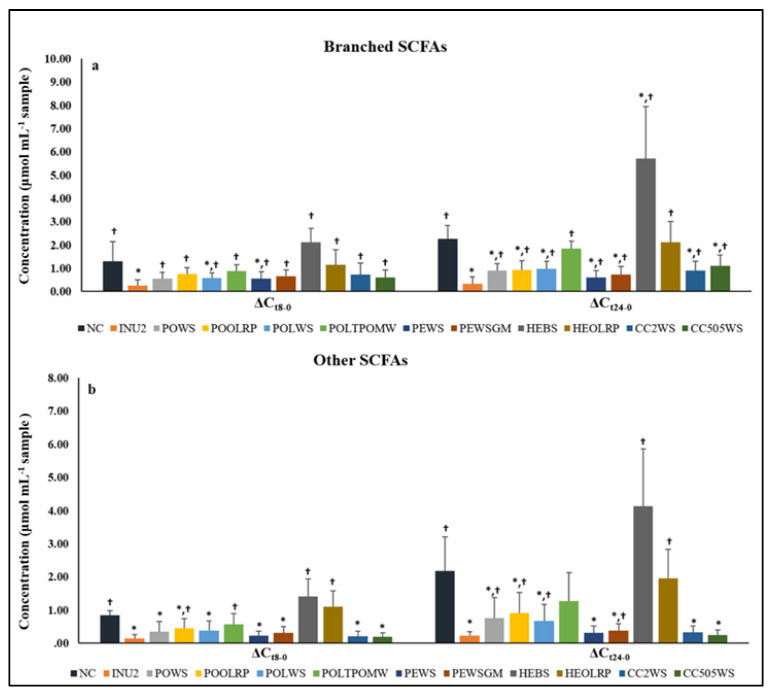
Differences (Δ) in concentrations (μmol mL^−1^) of (**a**) branched SCFAs (BSFAs) and (**b**) other SCFAs after 8-h fermentation (ΔCt8-0) and 24-h fermentation (ΔCt24-0) compared to baseline. Values are expressed as mean and SD, where ΔCt8-0 is defined as ‘Concentration t = 8 h minus Concentration t = 0 h’ and ΔCt24-0 is defined as ‘Concentration t = 24 h minus Concentration t = 0 h’ (for POLTPOMW and HEBS n = 4 - for CC2WS and CC505 n = 7); *: significant difference compared to NC (*p* < 0.05) (Mann-Whitney test or *t-test*); †: significant difference compared to INU2 (*p* < 0.05) (Mann-Whitney test or *t*-test).

Concentrations of total volatile fatty acids (VFAs) and SCFAs, so as molar ratios of SCFAs, at baseline and after 8-h and 24-h fermentation are presented in Table 4 and Table 5. Furthermore, Figure 2a–d and Figure 3a,b illustrate the mean differences of total VFAs (ΔTVFAs) and SCFAs concentration after 8 h and 24 h of fermentation.

Baseline (t = 0 h) average concentration of total VFAs was approximately 3.0 μmol mL^−1^ for all treatments and controls, reaching 67.0 and 95.0 μmol mL^−1^ after 8 h and 24 h of fermentation, respectively. Fermentation resulted in significantly higher levels of total VFAs in all treatments, compared to baseline (Table 4). Positive control (INU2) induced a significant increase in total VFAs concentration after 8-h and 24-h fermentation compared to NC, whereas all the tested mushrooms resulted in significantly higher total VFAs, compared to both NC and INU2 for the two time points. HEBS and HEOLRP reached the highest total VFAs concentration at the end of the fermentation process (approximately 130–140 μmol mL^−1^) (Table 4 and Figure 2a–d).

The fermentation process resulted in significant concentration increments of the major SCFAs (acetate, propionate, butyrate) in all treatments (Table 4 and Figure 2a–d). Baseline (t = 0 h) average concentration of acetate was approximately 1.5 μmol mL^−1^ for all treatments and controls, reaching 31.0 and 38.0 μmol mL^−1^ after 8 h and 24 h of fermentation, respectively. Acetate concentration in the positive control (INU2) was marginally higher compared to NC at 8-h (*p* = 0.050) and 24-h fermentation (*p* = 0.053) (Table 4), though a higher production rate was observed in INU2 at both time points (Figure 2a–d). All tested mushrooms demonstrated higher concentration (Table 4) and production rate of acetate compared to NC (Figure 2a–d). Furthermore, some of them (POOLRP, POLTPOMW, PEWS, PEWSGM, CC2WS and CC505WS) induced significantly higher levels of acetate compared to the positive control (INU2), detected after only 8 h of fermentation, whereas others (POWS, POLWS, HEBS) exhibited an analogous effect after 24 h of fermentation (Table 4).

Baseline (t = 0 h) average concentration of propionate was approximately 0.3 μmol mL^−1^ for all treatments and controls, reaching 11.5 and 16.0 μmol mL^−1^ after 8 h and 24 h of fermentation, respectively. Propionate concentration in positive control (INU2) paralleled that of NC at 8-h (*p* = 0.265) and 24-h fermentation (*p* = 0.390) (Table 4), though a higher change in propionate concentration was observed in INU2 at both time points compared to NC (Figure 2a–d). Nevertheless, all mushrooms induced significantly higher levels of propionate compared to both controls after 8 h and 24 h of fermentation, with HEBS exerting more intense changes.

Average concentration of butyrate was approximately 0.9 μmol mL^−1^ for all treatments and controls at baseline (t = 0 h), reaching 23.0 and 39.0 μmol mL^−1^ after 8 h and 24 h of fermentation, respectively. Though a greater change in baseline butyrate concentration was observed in INU2 compared to NC during the fermentation process (Figure 2a–d), no significant difference of mean butyrate levels was observed between controls at 8 h (*p* = 0.161) or 24 h of fermentation (*p* = 0.460) (Table 4). All tested mushrooms demonstrated a significantly higher concentration and production rate of butyrate compared to NC and INU2 after 8 h and 24 h of fermentation (Table 4 and Figure 2a–d), except for POLTPOMW compared to INU2 at 8 h (*p* = 0.130). A remarkable increase in butyrate concentration was observed in the case of HEOLRP (70 μmol mL^−1^) after 24-h fermentation compared to baseline levels, and 25 μmol mL^−1^ higher than butyrate levels detected in the case of other mushrooms exhibiting notable changes (e.g., HEBS, CC505WS, PEWS).

The fermentation process also resulted in significant higher levels of minor SCFAs, such as branched SCFAs (BSCFAs, sum of iso-butyrate, iso-valerate and iso-caproate) and other SCFAs (e.g., valerate, caproic acid, heptanoic acid) in all treatments compared to baseline (Table 4).

The average concentration of BSCFAs was approximately 0.08 μmol mL^−1^ at baseline (t = 0 h) for all treatments and controls, reaching 0.9 and 1.4 μmol mL^−1^ after 8 h and 24 h of fermentation, with the same values being estimated at 0.12, 0.6 and 1.1 μmol mL^−1^, respectively, in the case of other SCFAs. Branched SCFA production was significantly elevated after 8 h and 24 h of fermentation in the case of NC, while a similar pattern was detected in the case of some mushroom/substrate combinations (HEBS, HEOLRP, POLTPOMW). HEBS was the only treatment that induced a significantly greater change of BSCFAs concentration compared to both NC and INU2. On the contrary, the production of BSCFAs was rather limited in the case of the INU2 and *P. eryngii* mushrooms (PEWS, PEWSGM), with PEWS being the only treatment demonstrating marginally similar levels of BSCFAs after 24 h-fermentation compared to INU2 (*p* = 0.066). The rest of the tested mushrooms exhibited significantly higher concentration of BSCFAs than INU2, but lower than NC after 24 h of fermentation (Table 4). The production of the other SCFAs was also significantly elevated in the case of NC, with HEBS and HEORLP following a similar pattern; on the other hand, the production of other SCFAs was rather limited in the case of INU2, with *P. eryngii* (PEWS, PEWSGM) and *C. cylindracea* (CC2WS, CC505WS) mushrooms showing an analogous effect. *P. ostreatus* mushrooms had a rather intermediate effect between NC and INU2 (Table 4 and Figure 3a,b).

Molar ratios of SCFAs (i.e., % total VFA proportion of SCFAs) at baseline and after 8-h and 24-h fermentation are presented in Table 5. In the case of the negative control (NC), significant increments were recorded in molar ratio of BSCFAs and other SCFAs, presumably in expense of molar ratios of butyrate and propionate, but not acetate. Fermentation of prebiotic inulin (INU2) induced the highest molar ratio of acetate among treatments (approximately 60% of total VFAs produced) and a significant reduction in molar ratios of BSCFAs and other SCFAs; in addition, a reduced molar ratio of butyrate was observed at 24 h compared to 8-h fermentation.

*P. ostreatus* mushrooms (POLWS, POOLRP, POLWS, POLTPOMW) resulted in a significant higher molar ratio of propionate after 8 h of fermentation, with a rather stable molar ratio of acetate and a stable to decreased molar ratio of butyrate (Table 5). After 24 h of fermentation, a significant drop in molar ratio of acetate and an increase in molar ratio of butyrate were detected, while the molar ratio of propionate remained rather unchanged. Molar ratios of other SCFAs and BSCFAs were reduced during the fermentation of *P. ostreatus* mushrooms, with more limited BSCFAs-related effects in the case of POLTPOMW. Treatments involving *P. eryngii* mushrooms (PEWS, PEWSGM) also resulted in a significant higher molar ratio of propionate after 8 h of fermentation, which was maintained until the end of fermentation. Furthermore, a significant drop in molar ratio of acetate with a subsequent significant increase in molar ratio of butyrate (approximately 45% of total VFAs produced) was observed between 8 h and 24 h of fermentation of both mushrooms. Molar ratio of other SCFAs and BSCFAs was significantly reduced during the fermentation of *P. eryngii* mushrooms, with PEWS inducing a significant decrease in molar ratio of BSCFAs between all-time points of fermentation.

The use of *C. cylindracea* mushrooms (CC2WS, CC505WS) resulted, also, in a significant higher molar ratio of propionate after 8 h of fermentation, which was preserved until the end of fermentation. Furthermore, a significant drop in molar ratio of acetate with a subsequent significant increase in molar ratio of butyrate (approximately 43% of total VFAs produced) was observed between 8 h and 24 h fermentation of both mushrooms. The molar ratio of other SCFAs and BSCFAs was significantly reduced during the fermentation of both *C. cylindracea* strains (Table 5).

A more versatile behavior was observed when *H. erinaceus* mushrooms (HEBS, HEOLRP) were compared (Table 5). In detail, both treatments resulted in a significantly higher molar ratio of propionate after 8 h of fermentation, which was preserved until the end of fermentation, with HEBS exhibiting the highest final molar ratio of propionate among all treatments (approximately 25% of total VFAs produced). Mushrooms from both substrates induced a decrease in molar ratio of acetate during fermentation, with a more drastic effect noted in the case of HEOLRP. Furthermore, fermentation of HEOLRP resulted in a significantly increased and scalable response of molar ratio of butyrate, resulting in the highest values among all treatments at the end of the process (approximately 60% of total VFAs), an effect not observed in the case of HEBS. In fact, molar ratios of major SCFAs (acetate:propionate:butyrate) after 24 h fermentation were 35:25:35 for HEBS and 20:15:60 for HEOLRP. For minor SCFAs, 24 h fermentation of HEOLRP resulted in a decreased molar ratio of other SCFAs and -most likely- BSCFAs (*p* = 0.069) compared to the baseline, whereas HEBS induced a significant reduction in molar ratio of other SCFAs at 8-h fermentation with both molar ratios of other SCFAs or BSCFAs almost returning to baseline levels after 24-h fermentation.

Finally, comparison of the same mushroom strain in different substrates (POWS vs. POOLRP, POLWS vs. POLTPOMW, PEWS vs. PEWSGM, HEBS vs. HEOLRP) or of different strains of the same species cultivated in the same substrate (POWS vs. POLWS, CC2WS vs. CC505WS) resulted at a significantly higher production of BSCFAs in POLTPOMW and HEBS compared to POLWS and HEOLRP, respectively. Similarly, a trend for higher production of other SCFAs in HEBS compared to HEOLRP after 24 h of fermentation was observed, with no significant differences among treatments. The effects of HEBS and POLTPOMW detected on SCFAs profile (e.g., BSCFAs, other SCFAs, propionate) were also verified for the first four runs of the experiment with all substrates available (data not shown).

## 3. Discussion

Mushrooms contain a plethora of bioactive components, including polysaccharides, composed of glucose, mannose, galactose, fucose, arabinose, glucuronic acid and β-D-glucans, which are considered to have a beneficial effect on human health [33,34,35]. Although several in vitro gut models have been used to explore the role of dietary fibers on the gut microbiota, to the best of our knowledge, there are only a few studies examining the effects of edible mushrooms (i.e., lyophilized powder from the entire fruitbody) on the gut microbiota composition and/or metabolites production [27,29]. For this reason, the aim of this work was to examine the effects of edible mushrooms using an in vitro batch culture fermentation system, inoculated with fecal samples from apparently healthy subjects over 65 years old.

In elderly persons, alterations in the intestinal function are evident, such as increased mucosal membrane permeability, changes in immune function and microbial dysbiosis [26]. Studies have shown decreased intestinal levels of *Bacteroides* and *Bifidobacterium* spp. in the elderly populations, whereas an increase in facultative anaerobes, such as streptococci, enterococci and enterobacteria, is evident [36,37,38]. In recent years, several in vitro and in vivo approaches have attempted to reverse this effect through various dietary formulations, such as probiotics and prebiotics [39,40,41,42].

In our study, *Pleurotus* spp. and *C. cylindracea* mushrooms induced a significant bifidogenic effect, compared to the baseline. The same effect was further observed for POOLRP and CC505WS after 24 h of fermentation compared to NC. Recently Zhao, et al. [27] demonstrated that the in vitro fermentation of *P. ostreatus* and *P. eryngii* mushrooms promoted the growth of *Bifidobacterium* spp. This is in line also with several in vitro and animal-based data, which demonstrated a bifidogenic effect of *Pleurotus* spp. extracts, a phenomenon dependent on the variability among strains and on the chemical composition of growth substrates [43,44].

An increase in bifidobacteria is considered as a marker of intestinal health, and many studies have highlighted their beneficial effect on the prevention of colorectal cancer, colon regularity and acute diarrhea [45]. To the best of our knowledge, there are no published data demonstrating the impact of *C. cylindracea* mushrooms or extracts on the intestinal *Bifidobacterium* spp. levels. The potential lactogenic effect of polysaccharide extracts from *P. eryngii* has been reported in a few studies [44,46,47]. Our results demonstrated that *P. eryngii* mushrooms from both substrates induced a significant increase of lactobacilli compared to NC and baseline after 24-h fermentation, showing an analogous effect to the prebiotic inulin. In contrast, the in vitro fermentation of *P. ostreatus* and *P. eryngii* mushrooms did not favor the growth of lactobacilli Zhao, et al. [27]. Likewise, in a different in vitro model the fermentation of *P. eryngii* mushrooms with fecal inoculum from healthy donors, resulted in no remarkable change in *Lactobacillus* spp. levels, whereas the ratio of bifidobacteria/lactobacilli/Enterobacteriaceae remained stable during the fermentation process [29]. These contradictory results are probably due to intrinsic factors that influence the bioactive compounds content among strains of the same species, as it is already reported in basidiomycetes [48,49].

*F. prausnitzii* (*Ruminococcaceae*) and *E. rectale*/*Roseburia* spp. group (*Lachnospiraceae*) are two of the most dominant butyrate-producers [24]. The levels of the colonic butyrate-producing bacteria are related to host health, since diabetic populations, human colorectal cancer patients, elderly people and IBD patients are characterized by reduced levels of butyrate producers and increased levels of opportunistic pathogens. The *E. rectale*/*Roseburia* spp. group is abundant in the gut microbiota, whereas lower numbers appear (with parallel decrease in fecal butyrate concentration) when a high protein and low carbohydrate diet is followed by human volunteers [50]. Notably, in our study, 24-h fermentation of PEWSGM significantly increased the levels of both bacterial groups, whereas POWS, POOLRP, POLWS, PEWS, CC505WS had positive effects in *F. prausnitzii* population, i.e., a bacterium that has been related to anti-inflammatory properties [51]. On the contrary, these bacterial populations were significantly reduced when no carbohydrate source was provided (negative control).

The Prebiotic Index allows for the comparison of the prebiotic effect of different substrates [52]. It remains a useful tool and it has been applied in the prebiotic investigation of various natural substrates (e.g., almond skins) [53] or processed carbohydrates, such as inulin, fructooligosaccharides, polydextrose and isomaltooligosaccharides [54]. As shown in Figure 1a,b, all mushroom treatments exhibited positive prebiotic indexes after 24-h fermentation. The highest values were observed in *P. eryngii* (PEWS) due to its strong lactogenic effect and in *C. cylindracea* (CC505WS), due to its bifidogenic effect and decrease of *Bacteroides* spp. levels. The prebiotic inulin and the mushroom PEWS were the only substrates that had significantly increased PIs compared to NC.

SCFAs are mainly produced from carbohydrate fermentation and have been shown to contribute significantly to host health. The most common SCFAs in the colon (acetate, propionate, butyrate) reduce the luminal pH resulting in gut microbial alterations and growth inhibition of pH-sensitive pathogens, increase in mineral absorption and influence intestinal motility [18]. Many studies have demonstrated that the fermentation of prebiotic substrates results in increased production of SCFAs [55]. However, variations in the chemical structure of prebiotics are known to affect their utilization by the gut microbiota and, thus, SCFAs production [45]. Acetate, propionate and butyrate are present in the colon in an approximate molar ratio of 60:20:20, respectively, although the amount and relative proportion of each SCFA is depending on the substrate, the microbiota composition and gut transit time [56].

In our study, fermentation process resulted in significant concentration increments of the major SCFAs (acetate, propionate, butyrate) in all substrates. The fermentation of all mushroom-based substrates induced the production of acetate compared to NC for both time points (8 h, 24 h) (Table 4 and Figure 2a-d). Some of the treatments (POOLRP, POLTPOMW, PEWS, PEWSGM, CC2WS and CC505WS) induced significantly higher levels of acetate, compared to the prebiotic control INU2 after 8 h of fermentation, whereas others (POWS, POLWS, HEBS) exhibited an analogous effect after 24 h of fermentation. In the present study, a significant drop in molar ratio of acetate with a subsequent significant increase in molar ratio of butyrate was observed for *P. eryngii* and *C. cylindracea* mushrooms between 8 h and 24 h of fermentation, and the same effect was observed for *P. ostreatus* mushrooms after 24 h of fermentation.

In a recent in vitro fermentation study [27], *P. ostreatus* exhibited higher concentrations of total SCFAs, acetate, propionate and butyrate, compared to the baseline and the control; on the contrary, *P. eryngii* increased the concentrations of all SCFAs compared to the baseline, but they remained significantly lower than the control. Acetate is widely produced by different bacterial groups in the gut, while propionate and butyrate are more substrate-specific [57]. Bifidobacteria are considered as important acetate producers during fermentation of inulin-type fructans, and they participate in cross-feeding interactions with other gut bacterial groups resulting in the production of propionate and butyrate [58].

All mushrooms induced significantly higher levels of propionate, compared to both controls after 8 h and 24 h of fermentation, with HEBS exerting more intense changes. In addition, all tested mushrooms also resulted in a significantly higher molar ratio of propionate after 8 h of fermentation. This remarkable increase of propionate from HEBS fermentation could possibly be explained by the high substrate content in rhamnose and fucose [59,60]. In this study, the fermentation of *H. erinaceus* mushrooms (HEBS and HEOLRP) has not yielded a proportional increase in the tested bacterial populations known for their propionate production [50]. Intestinal bacteria may produce propionate via three different pathways, depending on the chemical structure of the available substrate and on their genetic background. The succinate pathway is widely distributed in Bacteroidetes and in some Firmicutes species. The acrylate pathway is more restricted within the gut microbes, whilst the propanediol pathway is activated specifically when deoxy sugars (e.g., fucose and rhamnose) enriched substrates are present. Members of the family *Lachnospiraceae*, e.g., *Blautia* spp., as well as *Ruminococcus* and *Roseburia* species, were found to follow this pathway [50].

Butyrate can be absorbed by the colonic mucosa, and serve as an energy substrate for intestinal epithelial cells, while it also exhibits anti-cancer, pro-apoptotic and anti-inflammatory properties [22,23]. All tested mushrooms demonstrated a significantly higher concentration and production rate of butyrate, compared to controls after 8 h and 24 h of fermentation (Table 4 and Figure 2a-d), whereas a remarkable increase was observed in the case of HEOLRP. In this study several mushroom treatments increased the *F. prausnitzii* population, whereas PEWSGM significantly increased the levels of both butyrate producing groups. On the contrary, in the case of HEOLRP, we did not notice an outstanding increase of the tested butyrate producers, concomitant to the observed butyrate production. Future studies will include the DNA (e.g., 16S rRNA) sequencing of the gut microbiota examined, in order to obtain more information about other, non-tested, butyrate producers.

Two main types of colonic microbial fermentation can be distinguished, i.e., the saccharolytic fermentation of carbohydrates, as most microorganisms preferentially use them, and proteolytic fermentation (when carbohydrate sources are depleted) [23]. Branched SCFAs (BSCFAs), e.g., isobutyric and isovaleric acid, are generated by fermentation of branched amino acids, valine, leucine and isoleucine, originating from undigestible protein reaching the colon [61]. In the present study, fermentation resulted in significantly higher levels of BSCFAs and other SCFAs in all treatments compared to baseline. Notably, the production of BSCFAs in the case of NC was significantly elevated after 8 h and 24 h of fermentation, due to proteolytic activity, whereas HEBS was the only treatment that induced a significantly greater change of BSCFAs concentration compared to both controls. In addition, PEWS was the only treatment demonstrating marginally similar levels of BSCFAs compared to INU2 after 24 h fermentation. The production of other SCFAs was also significantly elevated in the case of NC, with HEBS and HEORLP following a similar pattern.

In this study, several rich in β-glucan mushroom species were studied in terms of their prebiotic potential, most of them with rather unexplored effects in gut microbial dynamics. Furthermore, mushrooms obtained from various cultivation substrates deriving from agricultural and agro-industrial by-products were evaluated, offering new alternatives in their exploitation through their bioconversion into value-added products with functional properties. In our study, lyophilized powder from the entire mushrooms were examined, thus allowing the investigation of possible synergistic effects among different bioactive compounds. Most importantly, the study focused on apparently healthy subjects (fecal donors) over 65 years old, providing a model for exploring the beneficial effects of edible mushrooms in gut microbiota dynamics during aging process. The qPCR methodology, in combination with SCFA quantification, provided a detailed picture of microbial dynamics during fermentation process, while the calculation of Prebiotic Indexes made it possible to account for the physiological variability that characterizes the experimental process of the in vitro fermentation.

Nevertheless, in order to overcome the limitations mainly set by the extent of microbiome analysis, we are planning to further extend and validate our study through the application of other types of fermentation processes (e.g., continuous cultures) and gut microbiota sequencing.

## 4. Materials and Methods

### 4.1. Fungal Strains, Cultivation Substrates, Mushroom Production and Glucans Content

In the present study, six strains of *Pleurotus ostreatus* (IK 1123 and LGM 22), *Pleurotus eryngii* (LGAM 216), *Hericium erinaceus* (LGAM 4514) and *Cyclocybe cylindracea* (LGAM 951 and LGAM 961) were used. Pure cultures were established from material collected from various habitats in Greece, and are preserved in the fungal Culture Collection of Laboratory of General and Agricultural Microbiology (Agricultural University of Athens, Athens, Greece).

Cultivation substrates and conditions for mushroom production were as previously described [15,62,63]. Total and α-glucans were measured in quadruplicates by means of a commercial kit (Megazyme Ltd., Wicklow, Ireland); β-glucans content was calculated by subtraction of α-glucans from total glucans. Glucan content was expressed in % *w/w* of dry weight (d.w.). The treatments used in this study and their glucan content are presented in Table 1. Mushrooms were lyophilized and pulverized, before the in vitro fermentation process.

### 4.2. Faecal Donors’ Characteristics

Fecal donors were apparently healthy subjects (>65 yrs), meeting the following inclusion criteria: (a) body mass index (BMI) < 30 kg m^−2^, with no recent weight loss and extreme dietary behaviors; (b) no history of gastrointestinal disease, chronic constipation, chronic/acute diarrhea, autoimmune disease, coronary disease, liver and/or kidney malfunction; (c) no consumption of antibiotics two months before the study; and (d) no consumption of probiotics and/or prebiotics and/or dietary fiber supplements two weeks before the study.

Subjects completed a series of questionnaires in relation to sociodemographic parameters (including age, sex, marital status and education level), smoking habits and medical history. Evacuation characteristics were also recorded for the past 7 days prior to fecal sampling. Body weight and height were measured by a dietician on a levelled platform scale (SECA GmbH, Hamburg, Germany) and a wall-mounted stadiometer (SECA GmbH), to the nearest 0.1 kg and 0.5 cm, respectively. Body mass index (BMI) was calculated by dividing the weight (kg) by the height (m^2^) [64]. Dietary intake was evaluated through a 3-d food diary, and data were analyzed in terms of energy and nutrient intakes, using the Nutritionist Prο software (version 4.1.0.; Axxya Systems, Stafford, TX, USA). Physical activity assessment was performed by the International Physical Activity Questionnaire Short Form questionnaire validated for the Greek population [65]; duration of sedentary activity (sitting or resting, h wk^−1^) was also reported [64].

### 4.3. Ethical Standards

The study was conducted according to the guidelines laid down in the Declaration of Helsinki, and under the approval of the Bioethics Committee of Harokopio University, Athens, Greece (62-03/07/2018). Written informed consent was obtained from all fecal donors, prior to their inclusion in the study.

### 4.4. Fecal Sample Collection and In Vitro Static Batch Culture Fermentations

Fecal sample collection was performed as previously described [64]. In detail, participants were given a pre-weighed plastic container to collect and return their whole evacuation the next few days. Stool samples were weighted, homogenized and processed within two hours after defecation.

The in vitro static batch culture fermentation process was performed according to the protocol of Olano-Martin, et al. [66] and Rycroft, et al. [67] with slight modifications. The composition of the basal medium was further modified by reducing or excluding from the recipe some evidence-based ingredients with cytotoxic effect (e.g., hemin, Tween^®^ 80, resazurin) [68,69]. Based on previous testing, modified basal medium was comparable to standard basal medium in terms of in vitro fermentation potential and microbial dynamics (data not shown).

The modified basal medium consisted of the following ingredients (g L^−1^): peptone water (Merck KGaA, Darmstadt, Germany), 2.0; yeast extract (Merck KGaA), 2; NaCl, 0.10; K_2_HPO_4_, 0.04; KH_2_PO_4_, 0.04; MgSO_4_.7H_2_O, 0.01; CaCl_2_.2H_2_O, 0.01; NaHCO_3_, 2.0; L-cysteine HCl (Merck KGaA), 0.50; dehydrated bile (Oxgall^TM^, BD and Company, Sparks, MD, USA), 0.50; hemin (dissolved in some drops of NaOH 1.0 M) (Fluka, Sigma-Aldrich Chemie N.V., Zwijndrecht, Netherlands), 0.005 [69]; Tween^®^ 80 (Pancreac Quimica SA, Barcelona, Spain), 0.2 mL L^−1^ [68] and vitamin K1 (Fluka, Sigma-Aldrich Chemie GmbH, Buchs, Switzerland), 10 μL L^−1^. The medium was pH controlled at 7.0 with HCl 1.0 M, volumes of 72 mL were aliquoted into 100 mL vessels, sterilized at 121 °C for 15 min and transferred into the anaerobic chamber (BACTRON™ 1.5 Anaerobic Environmental Chamber, SHELLAB, Cornelius, OR, USA) for a 12 h overnight pre-reduction the day before the in vitro static batch culture fermentation process.

At the day of the in vitro experiment, 2% (*w/v*) of the lyophilized mushrooms powder was added in the basal medium aliquots. The documented prebiotic inulin was used as positive control for the fermentation procedure (2% *w/v*, INU2) (Orafti^®^ GR, BENEO-Orafti, Oreye, Belgium), whereas a negative control (NC; basal medium with no carbohydrate source) was also included in the experiment. A fecal slurry (20% *w/v*) was prepared in PBS pH 7.3 (8.0 g L^−1^ NaCl, 0.2 g L^−1^ KCl, 1.15 g L^−1^ Na_2_HPO_4_, 0.2 g L^−1^ KH_2_PO_4_) [70] and homogenized in a Stomacher^®^ paddle blender (Seward Laboratory Systems Inc., Bohemia, NY, USA) under normal speed (265 rpm) for approximately 20 s. From this slurry, 10% (*v/v*) inocula were transferred into the pre-reduced basal medium aliquots with the tested mushrooms or the controls. The static batch cultures were incubated for 24 h under anaerobic conditions at 37 °C. Samples were collected at baseline (0 h), 8 h and 24 h of fermentation, and stored at −80 °C until further analysis (gut microbiota and SCFAs profiling). Due to limited quantity of some lyophilized mushrooms, fewer runs were performed for POLTPOMW (n = 4), HEBS (n = 4), CC2WS (n = 7) and CC505WS (n = 7).

### 4.5. Gut Microbiota Analysis

Total bacterial load and selected members of gut microbiota were enumerated at baseline (0 h) and after 24 h fermentation by real-time quantitative PCR (qPCR) as previously described [64]. Primers and qPCR characteristics of gut microbiota analysis are presented in Table 6.

Quantitative real-time PCR based on SYBR Green I detection chemistry was used to characterize the gut microbiota using species-, genus- and group-specific primers targeting 16S rRNA genes of different bacterial groups and the KAPA SYBR^®^ Fast Master Mix (2×) Universal Kit (Kapa Biosystems Inc., Wilmington, MA, USA) (Table 6) [64]. PCR amplification and detection were performed in a LightCycler^®^ 2.0 Real-Time PCR System (Roche Diagnostics GmbH, Mannheim, Germany). PCR reactions (final volume 20 μL) were performed in duplicate, in LightCycler^®^ glass capillaries and contained 10 ng of each fecal DNA preparation (2 ng μL^−1^), 10 μL of KAPA kit, 200 nM of each primer, 0.25 μL of Bovine Serum Albumin (BSA 20 mg mL^−1^, New England Biolabs Inc, Hitchin, UK) for minimization of reagent abstraction on glass capillaries surface and 3.95 μL PCR-grade water. The thermal cycling conditions included an initial enzyme activation step at 95 °C for 3 min, followed by 45 cycles of DNA denaturation at 95 °C for 3 s, primer annealing at optimal annealing temperature for 20 s and extension at 72 °C for the minimum time required for data acquisition at 72 °C, according to instrument guidelines [template size(bp)/25]. For the confirmation of the specificity of the amplification products, the melting curve analysis was performed by slowly cooling the PCRs from 95 °C to 65 °C (0.1 °C s^−1^) with simultaneous measurement of the SYBR Green I signal intensity. Microbial quantification was based on standard curves of genomic DNA from reference strains with the LightCycler^®^ software version 4.1 (Roche Diagnostics GmbH). Data are expressed as log_10_ copies of 16S rRNA gene mL^−1^ of sample [64].

### 4.6. Prebiotic Indexes

Prebiotic Indexes (PIs) were calculated after 24 h of fermentation of the tested substrates, providing a useful tool for the comparison of prebiotic efficiency [66]. Calculation of PIs was based on quantification of bacteria (copies of 16S rRNA gene mL^−1^ of sample) and the following equation [52]:

$$\mathrm{PI}\;=\left(\frac{\mathrm{Bif}}{\mathrm{Total}}\right)-\left(\frac{\mathrm{Bac}}{\mathrm{Total}}\right)+\left(\frac{\mathrm{Lac}}{\mathrm{Total}}\right)-\left(\frac{\mathrm{Clos}}{\mathrm{Total}}\right)$$

where Bif is *Bifidobacterium* spp. numbers after 24 h of fermentation (t = 24)/numbers at baseline (t = 0), Bac is *Bacteroides* spp. numbers after 24 h of fermentation (t = 24)/numbers at baseline (t = 0), Lac is *Lactobacillus* group numbers after 24 h of fermentation (t = 24)/numbers at baseline (t = 0), Clos is *Clostridium perfringens* group numbers after 24 h of fermentation (t = 24)/numbers at baseline (t = 0) and Total is total bacteria numbers after 24 h of fermentation (t = 24)/numbers at baseline (t = 0). Based on the prebiotic index equation, an increase in the population of bifidobacteria and/or lactobacilli is assumed as a positive effect and an increase in bacteroides and/or clostridia is assumed as a negative effect. This prebiotic index equation offers the advantage of normalizing the bacterial population changes in relation to the initial microbial levels, accounting for the physiological variability that characterizes the experimental process of the in vitro fermentation [52].

### 4.7. Measurement of SCFAs

Capillary gas chromatography (GC) was applied for the determination of the short-chain fatty acids (SCFAs) concentrations of the in vitro static batch cultures, according to Mountzouris, et al. [79], as previously described [80]. In detail, samples (1 mL) were centrifuged at 13.000× *g* for 15 min at 4 °C, and 300 μL of the supernatant were stored at −80 °C until analysis. At the day of analysis, supernatants (300 μL) were vortexed and centrifuged at 13.000× *g* for 5 min at RT. Subsequently, 85 μL of each supernatant were mixed with 10 μL 2-ethyl-butyrate (20 mM, internal standard) (2-Ethyl butyric acid 99%, Sigma-Aldrich Corp., St. Louis, MO, USA) and 5 μL hydrochloric acid (HCl, 1 M). Samples of 1 μL were injected into a gas chromatographer (Agilent 6890 GC System, Agilent Technologies, Santa Clara, CA, USA) with a Supelco Nukol™ Capillary GC Column (size x I.D. 30 m × 0.25 mm, df 0.25 μm) (Sigma–Aldrich Corp., St. Louis, MO, USA). The concentrations of SCFAs were computed based on instrument calibration with SCFA standard mix (Supelco volatile acid standard mix, Sigma–Aldrich Corp., St. Louis, MO, USA). Total volatile fatty acids (VFAs) and individual SCFAs concentrations were expressed as μmol mL^−1^ of sample and molar ratios (% of VFAs) of acetate, propionate, butyrate, branched-chain SCFAs (BSCFAs; iso-butyrate, iso-valerate, iso-caproic acid) and other SCFAs (valerate, caproic acid and heptanoic acid) were also calculated. The production rates of total VFAs, major SCFAs (acetate, propionate, butyrate) and minor SCFAs (BSCFAs, other SCFAs) were further calculated by subtracting initial concentration (t = 0 h) of SCFAs from concentrations after 8 h or 24 h of fermentation (ΔC_t8-0_ or ΔC_t24-0_).

### 4.8. Statistical Analysis

The continuous variables are presented as mean values and standard deviation (SD) or median and interquartile range (Q1–Q3). Categorical variables are presented as frequencies (n, %). Normality of distribution of continuous variables was tested by the Shapiro-Wilk test. Bacterial levels (t = 0 h and t = 24 h) and SCFAs characteristics (t = 0 h, t = 8 h and t = 24 h) at each sampling time were compared by one-way ANOVA for parametric data and Kruskal Wallis test with Mann-Whitney test for non-parametric data and prospectively by repeated measures ANOVA (RM-ANOVA) for parametric data and the Friedman test for non-parametric data, after Bonferroni’s adjustment for multiplicity. Comparisons of bacterial levels and SCFAs characteristics into each treatment (NC, INU2, POWS, POOLRP, POLWS, POLTPOMW, PEWS, PEWSGM, HEBS, HEOLRP, CC2WS, CC505WS) between the different time periods (0 h, 8 h, 24 h) were performed by paired-samples T test for parametric data and Wilcoxon signed ranks test for non-parametric data. For comparisons of PIs and production rates of SCFAs (ΔC_t8-0_ or ΔC_t24-0_), parametric and non-parametric tests were applied (one-way ANOVA, Kruskal Wallis test, *t-test*, Mann-Whitney test). Correlation analysis between mean PIs and average glucans content of mushrooms were performed by the Spearman’s rank correlation test and linear regression analysis was based on log_10_-transformation of mean PIs values. The software program IBM^®^ SPSS^®^ Statistics version 21 was used for the statistical analysis of the results and the significance threshold was set at 5% (*p* < 0.05).

## 5. Conclusions

This research highlighted the potential of certain edible mushrooms rich in β- glucans as candidate prebiotics. Strains of the genera *Pleurotus* and *Cyclocybe* exhibited a beneficial influence on the composition of gut microbiota of apparently healthy subjects over 65 years old (increase of *Bifidobacterium* spp. and *F. prausnitzii* populations), whereas all mushrooms elicited increased molar ratio of propionate and butyrate after 24 h of fermentation. *H. erinaceus* mushrooms induced the highest changes in SCFAs production. The development of nutritional products for preventing pathological conditions and improving the quality of life is of utmost importance for the consumer’s health, for the elderly in particular. The application of in vitro, ex vivo and in vivo methodologies for evaluating the biological activities of selected edible mushrooms rich in β-glucans is expected to elucidate/establish the health-promoting properties of this type of bioactive compounds, and to pave the way for their use in novel functional food products.

## Figures and Tables

**Table 1:** Fungal strains, mushroom cultivation substrates and glucans content*.

Description	Abbreviation	Total Glucans (% *w/w*)	α-Glucans (% *w/w*)	β-Glucans (% *w/w*)
*Pleurotus ostreatus* IK 1123 in 100% wheat straw (WS, control substrate)	POWS	39.4 ± 1.4	8.7 ± 1.3	30.6 ± 1.9
*Pleurotus ostreatus* IK 1123 in olive pruning residues (OL)	POOLRP	38.5 ± 2.1	3.4 ± 0.2	35.1 ± 1.1
*Pleurotus ostreatus* LGM 22 in 100% wheat straw (control substrate)	POLWS	34.3 ± 2.7	6.6 ± 1.1	27.7 ± 2.4
*Pleurotus ostreatus* LGM 22 in OL:TPOMW (ratio 3:1, *w/w*) (TPOMW, two-phase olive mill wastes)	POLTPOMW	39.9 ± 0.8	4.9 ± 1.0	35.0 ± 0.4
*Pleurotus eryngii* LGAM 216 in 100% wheat straw (control substrate)	PEWS	46.6 ± 3.9	7.9 ± 1.3	38.7 ± 5.4
*Pleurotus eryngii* LGAM 216 in WS:GM (ratio 1:1, *w/w*) (GM, grape marc)	PEWSGM	49.7 ± 2.9	7.6 ± 1.4	42.2 ± 5.9
*Hericium erinaceus* LGAM 4514 in 100% beech sawdust (BS, control substrate)	HEBS	16.4 ± 0.1	1.0 ± 0.1	15.4 ± 0.2
*Hericium erinaceus* LGAM 4514 in olive pruning residues	HEOLRP	21.8 ± 0.9	1.1 ± 0.1	20.7 ± 0.3
*Cyclocybe cylindracea* LGAM 951 in 100% wheat straw (control substrate)	CC2WS	39.3 ± 1.7	6.1 ± 0.5	33.2 ± 1.9
*Cyclocybe cylindracea* LGAM 961 in 100% wheat straw (control substrate)	CC505WS	40.6 ± 2.9	3.4 ± 1.1	37.2 ± 3.8

* Values are expressed as mean and standard deviation (SD) of % *w/w* of dry weight (d.w.) (n = 4).

**Table 2:** Descriptive characteristics of fecal donors (n = 8).

Sociodemographic Parameters	
Sex (no. of males/females), n (%)	4/4 (50.0%/50.0%)
Age (years)	73.50 ± 5.88
Smoking (no. of smokers), n (%)	1 (12.5%)
Educational years	15.25 ± 4.71
Marital status (no. of married/widowed), n (%)	4/4 (50.0%/50.0%)
Medical history-Clinical evaluation	
Diagnosis/drug treatment for hypertension, n (%)	5 (62.5%)
Diagnosis/drug treatment for dyslipidemia, n (%)	2 (25.0%)
Drug treatment, n (%)	6 (75.0%)
Dietary supplements, n (%)	3 (37.5%)
Evacuation frequency (times·d^−1^)	1.00 (1.00–1.00)
Anthropometric measurements	
Body weight (kg)	63.55 (62.55–73.63)
Height (m)	1.64 ± 0.09
BMI (kg·m^−2^)	25.14 ± 3.16
Nutritional analysis-Physical activity	
Energy intake (kcal·d^−1^)	1585.99 ± 582.31
Carbohydrate (% of energy)	42.91 ± 6.68
Carbohydrate (g·d^−1^)	173.99 ± 69.47
Protein (% of energy)	18.10 ± 4.24
Protein (g·d^−1^)	69.83 ± 20.89
Fat (% of energy)	36.94 ± 5.54
Fat (g·d^−1^)	67.38 ± 30.73
SFA (g·d^−1^)	22.41 ± 9.60
MUFA (g·d^−1^)	22.83 (19.78–37.70)
PUFA (g·d^−1^)	8.52 (5.20–9.72)
Fiber (g·d^−1^)	14.89 ± 10.26
Total Physical Activity (MET-min·wk^−1^)	1333.38 ± 876.79
Sitting or resting time (h·wk^−1^)	34.13 ± 14.21
Moderate level of physical activity, n (%)	6 (75.0%)

Values are expressed as mean and SD for parametric or median and Q1-Q3 quartiles for nonparametric data; BMI: Body Mass Index; MET: Metabolic equivalent of task; MUFA: Monounsaturated Fatty Acids; PUFA: Polyunsaturated Fatty Acids; SFA: Saturated Fatty Acids.

**Table 3:** Fecal microbial quantification (quantitative PCR (qPCR); log_10_ copies of 16S rRNA gene mL^−1^ of sample) at baseline (t = 0 h) and after 24 h fermentation.

**Baseline (t = 0 h)**							
	Total Bacteria	*Lactobacillus* Group	*Bifidobacterium* spp.	*Bacteroides* spp.	*Clostridium perfringens* group	*Roseburia* spp.-*Eubacterium rectale*	*Faecalibacterium prausnitzii*
NC	10.11 (10.02–10.21)	6.13 (5.84–7.20)	8.79 (7.40–9.07)	9.48 ± 0.27	6.59 (6.35–6.75)	8.45 (8.17–8.78)	8.86 (8.60–9.06)
INU2	10.20 (10.08–10.30)	6.19 (5.84–7.32)	8.81 (7.38–9.04)	9.56 ± 0.24	6.62 (6.44–6.83)	8.53 (8.22–8.72)	8.97 (8.59–9.05)
POWS	9.99 ^†^ (9.94–10.12)	6.09 (5.82–7.20)	8.78 (7.31–9.02)	9.50 ± 0.21	6.44 (6.11–6.69)	8.20 (8.04–8.55)	8.76 (8.40–8.86)
POOLRP	9.96 *^,†^ (9.81–10.01)	5.98 (5.73–7.06)	8.71 (7.28–9.06)	9.40 ± 0.17	6.36 (6.08–6.84)	8.15† (8.05–8.49)	8.67 (8.40–8.82)
POLWS	10.14 (10.06–10.22)	6.12 (5.84–7.26)	8.74 (7.25–9.08)	9.59 ± 0.18	6.76 (6.51–6.91)	8.58 (8.26–8.74)	9.00 (8.64–9.13)
POLTPOMW	10.02 (9.89–10.15)	6.68 (6.24–7.28)	7.87 (6.80–8.87)	9.64 ± 0.31	6.86 (6.34–7.13)	8.60 (8.57–8.61)	8.79 (8.64–9.13)
PEWS	9.84 *^,†^ (9.67–9.88)	5.94 (5.60–7.04)	8.73 (7.29–8.97)	9.37 ± 0.22	6.24 (5.97–6.73)	8.00 *^,†^ (7.72–8.23)	8.53 *^,†^ (8.35–8.74)
PEWSGM	10.05 ^†^ (9.72–10.09)	6.03 (5.72–7.21)	8.71 (6.98–9.10)	9.51 ± 0.23	6.48 (6.21–6.76)	8.08 *^,†^ (7.96–8.51)	8.74 (8.42–8.87)
HEBS	10.10 (9.97–10.30)	6.46 (5.83–7.20)	7.90 (6.74–9.02)	9.45 ± 0.22	6.11*^,†^ (5.73–6.40)	8.32 (8.04–8.51)	8.70 (8.39–8.87)
HEOLRP	10.12 (10.02–10.18)	5.99 (5.74–7.21)	8.87 (7.54–9.15)	9.51 ± 0.29	6.36 (5.96–6.65)	8.33 (8.12–8.50)	8.84 (8.66–8.98)
CC2WS	9.89 *^,† ^ (9.79–9.96)	5.92 (5.43–6.60)	8.36 (7.06–8.92)	9.32 ± 0.28	5.83 *^,† ^ (5.22–6.12)	8.09 *^,† ^ (7.82–8.38)	8.52 *^,†^ (8.27–8.74)
CC505WS	10.11 ^†^ (9.81–10.15)	5.98 (5.61–6.89)	8.53 (7.26–9.06)	9.47 ± 0.30	6.12 *^,†^ (5.66–6.49)	8.53 (7.26–9.06)	8.30 (8.19–8.62)
**24-h Fermentation (t = 24 h)**							
	Total Bacteria	*Lactobacillus* Group	*Bifidobacterium* spp.	*Bacteroides* spp.	*Clostridium perfringens* Group	*Roseburia* spp.-*Eubacterium rectale*	*Faecalibacterium prausnitzii*
NC	9.98 ^†,a^ (9.86–10.06)	5.95 ^†^ (5.69–7.05)	8.73 ^†^ (7.34–9.16)	9.04 ± 0.38 ^†,a^	6.38 (6.03–6.69)	7.97 ^a ^ (7.09–8.15)	8.29 ^†,a^ (7.83–8.62)
INU2	10.28 * (10.14–10.32)	7.66 *^,a^ (7.03–9.00)	9.83 *^,a^ (8.11–9.96)	9.68 ± 0.23 *	6.42 (6.22–6.70)	8.15 ^a^ (7.91–8.50)	9.06 *^,a^ (8.66–9.15)
POWS	10.36 *^,a^ (10.14–10.40)	6.48 (5.87–7.59)	9.49 ^a^ (8.12–9.66)	9.53 ± 0.33 *	6.58 (6.25–6.92)	8.38 * (7.97–8.46)	9.06 *^,a^ (8.78–9.39)
POOLRP	10.38 *^,a^ (10.20–10.49)	6.56 (5.84–7.35)	9.49 *^,a^ (8.36–9.82)	9.61 ± 0.16 *^,a^	6.71 ^a^ (6.60–6.82)	8.33 * (8.01–8.47)	9.10 *^,a^ (8.97–9.32)
POLWS	10.39 *^,a^ (10.28–10.46)	6.59 (5.87–8.26)	9.55 ^a ^ (8.27–9.66)	9.62 ± 0.34 *	6.75 * (6.57–7.32)	8.50 *^,†^ (8.40–8.79)	9.21 *^,a^ (8.96–9.32)
POLTPOMW	10.13 (10.08–10.36)	7.53 (6.09–8.83)	8.65 (6.99–9.57)	9.46 ± 0.47 *	6.50 (6.44–6.74)	8.58 *^,†^ (8.34–8.72)	8.78 (8.54–9.03)
PEWS	10.29 *^,a^ (10.18–10.37)	8.58 *^,a^ (7.40–9.32)	9.29 ^a^ (8.08–9.54)	9.50 ± 0.32^*^	6.52 ^a^ (6.13–6.91)	7.99 ^a ^ (7.89–8.33)	9.02 *^,a^ (8.75–9.58)
PEWSGM	10.44 *^,†,a^ (10.30–10.50)	7.93 *^,a^ (6.81–9.21)	9.36 ^a^ (8.29–9.74)	9.69 ± 0.23^*^	6.72 (6.28–7.08)	8.51 *^,†,a^ (8.39–8.58)	9.18 *^,a^ (8.96–9.79)
HEBS	10.12 (9.78–10.18)	6.45 (5.86–7.97)	8.19 ^†^ (6.72–9.29)	9.54 ± 0.27^*^	6.09 (5.89–6.66)	8.01 (7.43–8.25)	8.49 (8.02–8.97)
HEOLRP	10.19 * (10.10–10.36)	6.29 ^† ^ (5.72–7.14)	9.13 ^†,a^ (7.60–9.47)	9.61 ± 0.27^*^	6.50 (6.18–6.80)	8.34 * (8.06–8.42)	8.79 * (8.47–9.15)
CC2WS	10.14 ^a^ (9.96–10.24)	6.23 ^a ^ (5.87–9.14)	9.38 ^a^ (7.64–9.88)	8.88 ± 0.45 ^†,a^	6.37 ^a ^ (5.67–6.52)	7.85 ^†,a ^ (7.52–8.14)	8.74 ^a ^ (8.48–9.00)
CC505WS	10.25 *^,a^ (10.07–10.35)	6.27 (5.75–9.22)	9.69 *^,a^ (7.84–9.83)	9.07 ± 0.52 ^†,a^	6.40 (5.92–6.62)	8.07 (7.90–8.25)	8.98 *^,a^ (9.00–9.24)
*P* overall	0.018	0.733	0.939	0.001	0.022	0.001	0.143

Values are expressed as mean and SD for parametric or median and Q1–Q3 quartiles for nonparametric data (for POLTPOMW and HEBS n = 4 - for CC2WS and CC505 n = 7; *: significant difference compared to NC (*p* < 0.05) at baseline (t = 0 h) or after 24-h fermentation (t = 24 h) (repeated measures ANOVA after Bonferroni’s adjustment for multiplicity or Friedman’s test); †: significant difference compared to INU2 (*p* < 0.05) at baseline (t = 0 h) or after 24-h fermentation (t = 24 h) (repeated measures ANOVA after Bonferroni’s adjustment for multiplicity or Friedman’s test); a: significant difference compared to baseline (paired-samples T test for parametric data and Wilcoxon signed ranks test for non-parametric data).

**Table 4:** Total volatile fatty acids (VFAs) and SCFAs concentrations (μmol mL^−1^ of sample) at baseline and after 8-h and 24-h fermentation.

**Baseline (t = 0 h)**						
**Concentrations (** **μmol mL^−1^** **)**						
	Total VFAs	Acetate	Propionate	Butyrate	BSCFAs	Other SCFAs
NC	3.80 ± 1.33 ^†^	1.55 (1.32–2.14)	0.43 (0.30–0.59)	1.14 ^† ^ (0.61–2.15)	0.10 (0.06–0.16)	0.11 (0.09–0.22)
INU2	2.59 ± 0.71 *	1.49 (0.81–1.65)	0.37 (0.21–0.45)	0.67 * (0.58–0.83)	0.10 (0.09–0.11)	0.10 (0.08–0.16)
POWS	3.12 ± 0.68	1.56 (1.13–1.69)	0.32 (0.16–0.53)	1.14 ^†^ (0.87–1.37)	0.07 ^†^ (0.06–0.08)	0.10 (0.09–0.20)
POOLRP	2.92 ± 0.68 *	1.48 1.05–1.80)	0.24 * (0.16–0.39)	0.97 ^†^ (0.90–1.26)	0.07 ^†^ (0.06–0.09)	0.11 (0.09–0.14)
POLWS	2.90 ± 0.97 *	1.44 (0.75–1.84)	0.26 (0.16–0.47)	0.99 ^†^ (0.82–1.36)	0.07 ^†^ (0.05–0.08)	0.10 (0.08–0.14)
POLTPOMW	2.94 ± 0.52	1.60 (1.24–1.80)	0.29 (0.22–0.46)	0.95 (0.68–1.04)	0.07 ^†^ (0.06–0.10)	0.10 (0.09–0.13)
PEWS	2.66 ± 0.72 *	1.33 (0.89–1.67)	0.25 * (0.18–0.39)	0.90 (0.63–0.99)	0.08 ^†^ (0.05–0.08)	0.10 (0.09–0.15)
PEWSGM	2.82 ± 0.78	1.55 (1.03–1.89)	0.28 * (0.19–0.41)	0.90 (0.66–0.98)	0.07 ^†^ (0.06–0.09)	0.10 (0.09–0.14)
HEBS	2.90 ± 0.62	1.52 (1.19–1.90)	0.32 (0.21–0.48)	0.88 (0.66–0.95)	0.08 (0.06–0.11)	0.12 (0.08–0.15)
HEOLRP	3.32 ± 0.93	2.15 ^†^ (1.36–2.68)	0.26 (0.21–0.42)	0.86 (0.62–0.93)	0.07 (0.06–0.09)	0.09 (0.08–0.12)
CC2WS	2.80 ± 0.75 *	1.48 (1.00–1.83)	0.28 (0.21–0.48)	0.83 (0.51–1.06)	0.08 (0.06–0.09)	0.11 (0.08–0.15)
CC505WS	2.60 ± 0.62 *	1.43 (0.88–1.65)	0.30 (0.21–0.41)	0.82 (0.62–0.89)	0.08 (0.06–0.09)	0.10 (0.10–0.13)
**8-h Fermentation (t = 8 h)**						
**Concentrations (** **μmol mL^−1^** **)**						
	Total VFAs	Acetate	Propionate	Butyrate	BSCFAs	Other SCFAs
NC	20.32 ± 4.57 ^†,a^	11.31 ^a^ (8.40–13.29)	1.96 ^a^ (1.71–3.69)	4.38 ^a^ (3.87–5.35)	1.27 ^†,a^ (0.66–2.25)	0.98 ^†,a^ (0.87–1.15)
INU2	41.77 ± 12.51 *^,a^	22.41 ^a^ (19.08–27.55)	4.15 ^a^ (3.02–6.26)	11.01 ^a^ (8.67–13.90)	0.25 *^,a^ (0.21–0.44)	0.29 *^,a^ (0.17–0.36)
POWS	66.43 ± 16.33 *^,†,a^	31.21 *^,a^ (22.93–38.89)	9.34 *^,†,a^ (7.56–14.24)	26.21 *^,†,a^ (17.20–28.04)	0.62 *^,a^ (0.36–0.83)	0.41 *^,a^ (0.29–0.67)
POOLRP	77.53 ± 13.90 *^,†,a^	36.13 *^,†,a^ (34.86–41.51)	11.90 *^,†,a^ (9.84–16.50)	26.40 *^,†,a^ (20.33–29.90)	0.75 ^†,a^ (0.69–0.89)	0.54 *^,†,a^ (0.37–0.83)
POLWS	67.66 ± 11.90 *^,†,a^	32.49 *^,a^ (27.15–37.04)	10.62 *^,†,a^ (9.90–13.66)	25.00 *^,†,a^ (18.50–26.36)	0.58 *^,†,a^ (0.51–0.78)	0.47 *^,†,a^ (0.29–0.76)
POLTPOMW	71.10 ± 12.02 *^,†,a^	36.19 *^,†,a^ (33.44–38.13)	12.77 *^,†,a^ (11.06–18.89)	21.24 *^,a^ (12.47–24.28)	0.86 ^†,a^ (0.75–1.23)	0.70 ^†,a^ (0.34–0.97)
PEWS	73.68 ± 19.96 *^,†,a^	35.19 *^,†,a^ (28.88–41.37)	10.82 *^,†,a^ (7.54–17.92)	25.59 *^,†,a^ (21.29–32.67)	0.62 *^,a^ (0.36–0.79)	0.35 *^,a^ (0.22–0.46)
PEWSGM	81.16 ± 24.52 *^,†,a^	36.69 *^,†,a^ (29.40–51.05)	10.75 *^,†,a^ (9.20–18.32)	28.51 *^,†,a^ (15.61–37.37)	0.86 ^†,a^ (0.42–0.89)	0.40 *^,a^ (0.28–0.58)
HEBS	95.81 ± 21.96 *^,†,a^	32.40 *^,a^ (28.36–33.84)	23.08 *^,†,a^ (13.49–29.10)	42.97 *^,†,a^ (19.49–53.54)	2.11 ^†,a^ (1.69–2.80)	1.74 ^†,a^ (0.93–1.89)
HEOLRP	89.70 ± 20.70 *^,†,a^	31.84 *^,a^ (25.12–36.75)	17.66 *^,†,a ^ (9.47–23.41)	36.14 *^,†,a^ (28.32–60.43)	1.05 ^†,a^ (0.70–1.46)	1.25 ^†,a ^ (0.94–1.59)
CC2WS	68.56 ± 16.26 *^,†,a^	30.75 *^,†,a^ (28.73–43.57)	8.63 *^,†,a ^ (6.90–15.27)	21.92 *^,†,a^ (16.14–26.49)	0.55 ^†,a^ (0.51–0.98)	0.30 *^,a^ (0.20–0.45)
CC505WS	68.12 ± 13.63 *^,†,a^	32.26 *^,†,a^ (27.92–38.39)	8.73 *^,†, a^ (7.52–14.72)	21.82 *^,†,a^ (18.77–25.93)	0.75 *^,†,a^ (0.42–0.80)	0.26 *^,a^ (0.23–0.40)
**24-h Fermentation (t = 24 h)**						
	**Concentrations (** **μmol mL^−1^** **)**					
	Total VFAs	Acetate	Propionate	Butyrate	BSCFAs	Other SCFAs
NC	27.41 ± 5.27 ^†,a,b^	13.19 ^a,b^ (11.79–17.55)	2.90 ^a^ (2.35–3.28)	5.77 ^a,b^ (5.38–5.94)	2.33 ^†,a,b^ (1.97–2.94)	2.16 ^†,a,b ^ (1.83–2.67)
INU2	47.58 ± 14.06 *^,a^	28.28 ^a^ (20.05–32.92)	4.81 ^a^ (3.77–5.93)	10.74 ^a^ (7.60–18.65)	0.37 *^,a^ (0.32–0.46)	0.36 *^,a^ (0.24–0.51)
POWS	101.10 ± 15.73 *^,†,a,b^	40.32 *^,†,a,b^ (39.42–51.55)	14.04 *^,†,a,b^ (11.54–19.78)	41.66 *^,†,a,b^ (34.82–45.80)	0.93 *^,†,a ^ (0.70–1.29)	0.76 *^,†,a,b^ (0.50–1.06)
POOLRP	105.32 ± 19.75 *^,†,a,b^	45.56 *^,†,a,b^ (40.94–50.31)	15.03 *^,†,a,b^ (12.80–23.32)	42.17 *^,†,a,b^ (33.37–43.71)	0.90 *^,†,a ^ (0.65–1.21)	0.97 *^,†,a,b^ (0.67–1.31)
POLWS	94.45 ± 15.41 *^,†,a,b^	34.25 *^,a,b^ (31.13–42.87)	13.81 *^,†,a,b^ (12.37–21.63)	42.27 *^,†,a,b^ (32.58–44.15)	1.15 *^,†,a,b^ (0.76–1.30)	0.73 *^,†,a,b^ (0.45–0.89)
POLTPOMW	107.44 ± 8.88 *^,†,a,b^	46.52 *^,†,a,b ^ (45.07–50.57)	20.43 *^,†,a,b ^ (16.00–24.83)	37.71 *^,†,a,b^ (31.31–39.98)	1.96 ^†,a,b ^ (1.60–2.20)	1.44 ^†^ (0.56–2.15)
PEWS	98.32 ± 17.41 *^,†,a.b^	35.51 *^,†,a^ (31.17–47.60)	14.51 *^,†,a^ (10.80–19.70)	45.39 *^,†,a,b^ (44.67–46.11)	0.61 *^,a^ (0.50–0.84)	0.39 *^,a^ (0.25–0.58)
PEWSGM	103.18 ± 14.30 *^,†,a,b^	41.03 *^,†,a ^ (31.12–50.50)	16.46 *^,†,a ^ (10.58–20.58)	46.56 *^,†,a,b^ (33.08–52.68)	0.84 *^,†,a ^ (0.48–1.10)	0.46 *^,a ^ (0.37–0.62)
HEBS	141.08 ± 15.28 *^,†,a,b^	51.40 *^,†,a,b^ (40.02–53.88)	35.26 *^,†,a,b ^ (28.34–38.74)	50.51 *^,†,a,b ^ (32.60–62.29)	5.41 ^†,a,b ^ (3.99–8.02)	4.45 ^†,a,b ^ (2.50–5.80)
HEOLRP	133.45 ± 15.58 *^,†,a,b^	31.59 *^,a^ (24.99–39.75)	20.95 *^,†,a,b ^ (17.96–30.30)	80.90 *^,†,a,b^ (50.12–89.47)	1.99 ^†,a,b ^ (0.70–1.46)	2.17 ^†,a,b^ (1.43–2.64)
CC2WS	102.71 ± 17.32 *^,†,a,b^	38.92 *^,†,a,b^ (31.41–51.13)	15.13 *^,†,a,b^ (14.28–25.54)	47.17 *^,†,a,b^ (29.88–49.09)	0.85 *^,†,a^ (0.62–1.26)	0.44 *^,a,b^ (0.25–0.61)
CC505WS	110.64 ± 14.39 *^,†,a,b^	43.06 *^,†,a,b^ (34.91–48.64)	17.26 *^,†,a,b^ (13.12–28.97)	46.11 *^,†,a,b^ (42.77–50.71)	1.16 *^,†,a,b^ (0.79–1.55)	0.36 *^,a^ (0.20–0.51)
*p* overall	<0.001	0.015	0.006	<0.001	<0.001	<0.001

Values are expressed as mean and SD for parametric or median and Q1–Q3 quartiles for nonparametric data (for POLTPOMW and HEBS n = 4 - for CC2WS and CC505 n = 7); *: significant difference compared to NC (*p* < 0.05) at baseline (t = 0 h), after 8-h fermentation (t = 8 h) or after 24-h fermentation (t = 24 h) (repeated measures ANOVA after Bonferroni’s adjustment for multiplicity or Friedman’s test); †: significant difference compared to INU2 (*p* < 0.05) at baseline (t = 0 h), after 8-h fermentation (t = 8 h) or after 24-h fermentation (t = 24 h) (repeated measures ANOVA after Bonferroni’s adjustment for multiplicity or Friedman’s test); a: significant difference compared to baseline (paired samples *t-test* or Wilcoxon for non-parametric); b: significant difference compared to 8-h fermentation (paired samples *t-test* for parametric or Wilcoxon signed for non-parametric).

**Table 5:** Molar ratios (%) of SCFAs at baseline and after 8 h and 24 h fermentation.

**Baseline (t = 0 h)**					
**Molar Ratio (%)**					
	Acetate	Propionate	Butyrate	BSCFAs	Other SCFAs
NC	46.20 (41.82–54.41)	11.70 (11.29–15.87)	28.83 (21.56–42.37)	2.14 ^†^ (1.90–5.07)	2.81 (2.15–7.68)
INU2	50.68 (46.30–53.72)	13.05 (10.89–14.81)	26.29 (21.11–32.42)	3.56 * (3.18–5.32)	4.59 (2.84–7.95)
POWS	46.34 ^†^ (41.52–48.37)	8.95 *^,† ^ (7.28–17.12)	38.07 ^†^ (27.59–45.55)	2.17 ^†^ (1.99–2.35)	4.51 (2.60–5.72)
POOLRP	48.67 (45.64–51.55)	7.95 *^,†^ (7.41–11.01)	35.94 ^†^ (29.02–42.04)	2.48 ^†^ (2.02–2.69)	4.02 (2.68–6.05)
POLWS	44.91 ^†^ (40.37–49.66)	8.63 *^,†^ (8.17–11.75)	39.15 *^,† ^ (34.70–41.18)	2.32 ^†^ (1.92–2.92)	4.17 (2.65–5.95)
POLTPOMW	53.23 (50.29–54.05)	9.56 (9.04–13.75)	29.25 (26.65–34.85)	2.07 ^†^ (1.84–4.11)	3.16 (2.68–5.49)
PEWS	50.19 (45.62–51.99)	9.52 *^,†^ (8.96–10.86)	31.83 (28.39–37.69)	2.51 ^†^ (2.15–2.84)	4.64 (2.60–6.56)
PEWSGM	53.32 * (49.54–55.39)	9.51 *^,†^ (8.60–10.84)	30.90 (28.02–33.52)	2.66 ^†^ (1.95–3.05)	4.03 (2.69–5.10)
HEBS	52.54 (50.72–54.59)	10.82 (8.97–13.68)	28.17 (26.54–31.40)	2.45 (1.94–4.69)	4.54 (2.44–6.49)
HEOLRP	61.00 *^,† ^ (58.26–63.29)	8.45 *^,†^ (7.41–9.90)	24.10 * (22.74–27.69)	2.15 ^†^ (1.74–2.64)	2.97 (2.22–4.01)
CC2WS	52.94 * (50.32–55.49)	12.04 (9.67–12.69)	29.30 (24.37–30.04)	2.66 (2.00–3.43)	3.66 (2.86–5.67)
CC505WS	50.72 (46.01–51.65)	11.75 (10.59–12.44)	31.80 (29.85–32.22)	3.14 (2.00–3.78)	4.14 (2.98–5.85)
**8-h Fermentation (t = 8 h)**					
**Molar Ratio (%)**					
	Acetate	Propionate	Butyrate	BSCFAs	Other SCFAs
NC	52.73 (50.42–60.20)	11.36 (9.36–14.29)	22.63 ^a^ (20.40–25.86)	6.30 ^† ^ (3.95–8.88)	4.91 ^†^ (4.03–5.34)
INU2	59.38 ^a^ (50.88–64.45)	8.96 (8.82–15.10)	25.40 (19.33–34.05)	0.65 *^,a^ (0.45–1.38)	0.62 *^,a^ (0.37–1.13)
POWS	46.89 *^,† ^ (45.82–53.30)	14.96 *^,†,a^ (13.50–19.14)	34.93 *^,† ^ (31.98–39.32)	0.93 *^,a^ (0.60–1.37)	0.62 *^,a^ (0.36–1.38)
POOLRP	49.66 (45.82–53.30)	15.29 *^,†,a^ (14.60–19.95)	31.75 * (28.87–37.07)	0.96 *^,a^ (0.87–1.40)	0.66 *^,a^(0.45–1.27)
POLWS	47.58 *^,†^ (39.72–52.87)	16.70 *^,†,a^ (14.73–18.42)	32.80 * (29.31–38.77)	0.94 *^,a ^ (0.79–1.15)	0.70 *^,a^ (0.37–1.37)
POLTPOMW	50.26 (45.94–57.58)	19.36 *^,†,a ^ (17.02–22.98)	29.03 (19.73–31.08)	1.32 *^,† ^ (1.09–1.56)	0.99 *^,a ^ (0.43–1.64)
PEWS	47.86 ^†^ (42.29–53.56)	15.41 ^a^ (12.32–17.71)	33.18 * (31.89–39.53)	0.77 *^,a^ (0.62–1.02)	0.43 *^,a^ (0.30–0.83)
PEWSGM	46.42 ^†^ (44.03–58.02)	15.90 *^,†,a^ (12.83–17.61)	34.43 * (27.07–41.08)	0.78 *^,a^ (0.60–1.24)	0.53 *^,a^ (0.33–0.77)
HEBS	34.56 *^,† ^ (24.70–44.95)	24.18 *^,†,a ^ (15.72–28.08)	43.59 *^,†^ (25.55–46.70)	2.24 ^†^ (2.16–2.47)	1.85 ^†,a^ (0.87–2.39)
HEOLRP	36.93 *^,†,a^ (25.38–44.23)	18.14 *^,†,a^ (12.87–23.03)	44.56 *^,†,a ^ (34.01–53.36)	1.15 *^,†,a^ (0.91–1.78)	1.40 *^,†,a^ (0.94–2.03)
CC2WS	50.00 (43.03–60.96)	14.97 ^a ^ (11.46–17.31)	27.95 (26.08–34.19)	0.86 *^,a^ (0.79–1.19)	0.34 *^,a^ (0.31–0.76)
CC505WS	47.93 ^†^ (44.30–55.33)	14.61 ^a^ (12.39–18.38)	32.37 * (26.51–34.42)	0.89 *^,a^ (0.69–1.49)	0.36 *^,a^ (0.25–0.66)
**24-h Fermentation (t = 24 h)**					
**Molar Rratio (%)**					
	Acetate	Propionate	Butyrate	BSCFAs	Other SCFAs
NC	49.74 (45.86–56.75)	9.85 ^a^ (9.34–10.93)	19.85 ^a^ (18.89–23.29)	8.38 ^†,a^ (7.92–9.59)	7.34 ^†,b^ (6.41–8.71)
INU2	60.56 ^a,b^ (53.61–68.09)	8.74 (7.58–15.56)	23.16 ^b^ (18.02–33.35)	0.63 *^,a^ (0.55–1.51)	0.79 *^,a^ (0.42–1.13)
POWS	43.42 *^,†,b^ (38.20–47.77)	13.54 *^,a ^ (12.36–18.99)	40.67 *^,†,b^ (34.42–45.00)	0.91 *^,a ^ (0.64–1.37)	0.83 *^,a ^ (0.41–1.27)
POOLRP	44.23 ^†,b^ (40.94–50.11)	15.76 *^,†,a^ (13.62–17.83)	36.57 *^,†,b^ (31.91–40.41)	0.90 *^,a^ (0.61–1.29)	0.93 *^,a^ (0.55–1.55)
POLWS	40.96 *^,†,b^ (34.42–44.62)	15.40 *^,†,b^ (13.70–20.78)	42.43 *^,†,b^ (34.62–46.90)	1.34 *^,a^ (0.70–1.49)	0.77 *^,a^ (0.45–1.10)
POLTPOMW	44.69 ^†,a,b^ (41.51–46.43)	19.00 *^,†,a ^ (16.01–21.62)	34.31 ^b^ (30.58–36.42)	1.85 ^†^ (1.39–2.21)	1.34 *^,a^ (0.48–2.16)
PEWS	36.54 *^,†,a,b^ (30.43–46.46)	14.80 *^,a ^ (11.35–17.74)	45.21 *^,†,a,b^ (37.14–56.21)	0.60 *^,a,b^ (0.51–0.93)	0.34 *^,a ^ (0.24–0.65)
PEWSGM	38.92 *^,†,a,b ^ (33.40–46.16)	15.56 *^,†,a ^ (11.71–18.44)	45.15 *^,†,a,b^ (34.44–52.52)	0.76 *^,a ^ (0.48–1.00)	0.46 *^,a^ (0.34–0.60)
HEBS	35.84 *^,†,a^ (26.89–41.64)	24.78 *^,†,a,b^ (20.15–27.92)	35.10 * (25.41–40.94)	3.91 ^†^ (3.08–5.10)	3.17 ^†,b^ (1.66–4.41)
HEOLRP	20.49 *^,†,a,b^ (19.59–34.32)	14.48 *^,†,a ^ (13.28–23.42)	60.51 *^,†,a,b ^ (39.30–63.66)	1.52 *^,† ^ (1.06–2.47)	1.74 *^,†,a^ (0.96–1.91)
CC2WS	42.52 *^,†,a,b^ (29.66–43.38)	16.49 *^,†,a^ (13.31–16.65)	43.00 *^,†,a,b ^ (35.65–47.00)	0.92 *^,a^ (0.66–1.52)	0.37 *^,a ^ (0.22–0.65)
CC505WS	40.39 *^,†,a,b^ (34.37–42.31)	16.18 *^,†,a ^ (13.18–20.97)	43.16 *^,†,a,b ^ (37.21–47.54)	1.14 *^,a^ (0.65–1.45)	0.26 *^,†,a^ (0.21–0.50)
*p* overall	<0.001	0.272	0.004	<0.001	0.010

Values are expressed as mean and SD for parametric or median and Q1–Q3 quartiles for nonparametric data (for POLTPOMW and HEBS n = 4 - for CC2WS and CC505 n = 7); *: significant difference compared to NC (*p* < 0.05) at baseline (t = 0 h), after 8-h fermentation (t = 8 h) or after 24-h fermentation (t = 24 h) (repeated measures ANOVA after Bonferroni’s adjustment for multiplicity or Friedman’s test); †: significant difference compared to INU2 (*p* < 0.05) at baseline (t = 0 h), after 8-h fermentation (t = 8 h) or after 24-h fermentation (t = 24 h) (repeated measures ANOVA after Bonferroni’s adjustment for multiplicity or Friedman’s test); a: significant difference compared to baseline (Paired Samples *t-test* or Wilcoxon for non-parametric); b: significant difference compared to 8-h fermentation (Paired Samples *t-test* for parametric or Wilcoxon signed for non-parametric).

**Table 6:** Primers and qPCR characteristics of gut microbiota analysis (adapted by Mitsou, et al. [64]).

Target	Primer	Primer Sequence (5′-3′)	Annealing Temperature	Product Size	Reference Strains	References
Total Bacteria (Universal)	Forward Reverse	TCCTACGGGAGGCAGCAGT GGACTACCAGGGTATCTAATCCTGTT	60 °C	466 bp	*Bacteroides fragilis* MM44 (ATCC 25285)	[71]
*Lactobacillus* group	Forward Reverse	AGCAGTAGGGAATCTTCCA CACCGCTACACATGGAG	58 °C	341 bp	*Lactobacillus gasseri* DSM 20243	[72]
*Bifidobacterium* spp.	Forward Reverse	TCGCGTCYGGTGTGAAAG CCACATCCAGCRTCCAC	58 °C	243 bp	*Bifidobacterium bifidum* DSM 20456	[72]
*Bacteroides* spp.	Bac303F Bfr-Fmrev	GAAGGTCCCCCACATTG CGCKACTTGGCTGGTTCAG	60 °C	103 bp	*Bacteroides fragilis* MM44 (ATCC 25285)	[73]
*Clostridium perfringens* group	CPF CPR	ATGCAAGTCGAGCGATG TATGCGGTATTAATCTCCCTTT	55 °C	120 bp	*Clostridium perfringens* ATCC 13124	[74]
*Roseburia* spp.- *Eubacterium rectale*	RrecF Rrec630mR	GCGGTRCGGCAAGTCTGA CCTCCGACACTCTAGTMCGAC	60 °C	81 bp	*Roseburia intestinalis* DSM 14610	[75]
*Faecalibacterium prausnitzii*	FPR-2FFprau645R	GGAGGAAGAAGGTCTTCGG AATTCCGCCTACCTCTGCACT	60 °C	248 bp	*Faecalibacterium prausnitzii* DSM 17677	[76,77,78]

## Supplementary Materials

The following are available online, Figure S1a,b: Prebiotic Indexes (PIs) of the tested mushrooms and controls after 24 h fermentation for the first 4 runs of the in vitro fermentation experiment, Table S1: Prebiotic Indexes (PIs) per subject for each one of the treatments included in this study, Figure S2a–d: Linear regression analysis of log_10_-transformed mean Prebiotic Indexes (PIs) of the tested mushrooms with average total glucan content (a,c) and average β-glucan content (b,d) for all available data (a,b) and for the first 4 runs (c,d) of the in vitro fermentation experiment.
