# Supplementary material for: Effects of Rich in Β-Glucans Edible Mushrooms on Aging Gut Microbiota Characteristics: An In Vitro Study
Source: Molecules. 2020 Jun 18;25(12):2806. doi: 10.3390/molecules25122806 (PMC7355846; doi:10.3390/molecules25122806)
Supplement: Supplementary file 1 [file molecules-25-02806-s001.pdf]

molecules

Supplementary Information

Effects of rich in β-glucans edible mushrooms on aging gut microbiota characteristics: an *in vitro* study

Evdokia K. Mitsou [1], Georgia Saxami [1], Emmanuela Stamoulou [1], Evangelia Kerezoudi [1], Eirini Terzi [1], Georgios Koutrotsios [2], George Bekiaris [2], Georgios I. Zervakis [2], Konstantinos C. Mountzouris [3], Vasiliki Pletsa [4], Adamantini Kyriacou [1,*]

1 Department of Nutrition and Dietetics, Harokopio University, Athens, Greece; emitsou@hua.gr (E.K.M.); gsaxami@hua.gr (G.S.); dp4421749@hua.gr (E.S.); dp4421804@hua.gr (E.K.); ds214143@hua.gr (E.T.)
2 Laboratory of General and Agricultural Microbiology, Department of Crop Science, Agricultural University of Athens, Athens, Greece; georgioskoutrotsios@gmail.com (G.K.); giorgosbekiaris@yahoo.gr (G.B.); zervakis@aua.gr (G.I.Z.)
3 Department of Nutritional Physiology and Feeding, Agricultural University of Athens, Athens, Greece; kmountzouris@aua.gr (K.C.M.)
4 Institute of Chemical Biology, National Hellenic Research Foundation, Athens, Greece; vpletsa@eie.gr (V.P.)
* Correspondence: mkyriacou@hua.gr (A.K.); Tel.: +30-210-9549142

Table s1. Prebiotic Indexes (PIs) per subject for each one of the treatments included in this study.

	PIs/subject							
**Treatment**	**no.1**	**no.2**	**no.3**	**no.4**	**no.5**	**no.6**	**no.7**	**no.8**
**NC**	2.75	2.35	-0.83	5.42	1.06	-34.65	1.03	0.02
**INU2**	1235.98	11.88	1.01	77.65	19.77	7.84	49.72	14.46
**POWS**	62.99	1.53	-6.70	29.16	2.18	0.24	1.30	-0.78
**POOLRP**	16.51	3.46	-1.15	12.97	1.75	-0.21	0.50	0.84
**POLWS**	233.92	3.38	-2.93	19.55	3.68	0.85	0.64	0.18
**POLTPOMW**	52.68	1.51	-1.47	34.36	-	-	-	-
**PEWS**	2097.93	66.24	0.55	113.83	310.44	23.16	494.24	0.59
**PEWSGM**	446.21	1.86	-0.26	62.04	430.84	-1.09	172.96	1.06
**HEBS**	5.51	-0.52	-2.71	13.33	-	-	-	-
**HEOLRP**	6.02	1.59	0.16	16.74	0.19	-0.79	-1.34	-0.78
**CC2WS**	260.64	9.05	-0.56	484.74	8.39	1.79	-2.07	-
**CC505WS**	2705.53	4.77	-0.01	149.65	3.55	2.75	-3.61	-

POWS: *Pleurotus ostreatus* IK 1123 in 100% wheat straw (WS, control substate); POOLRP: *Pleurotus ostreatus* IK 1123 in olive pruning residues (OL); POLWS: *Pleurotus ostreatus* LGM 22 in 100% wheat straw (control substate); POLTPOMW: *Pleurotus ostreatus* LGM 22 in substate OL:TPOMW (ratio 3:1,w/w) (TPOMW: two-phase olive mill wastes); PEWS: *Pleurotus eryngii* LGAM 216 in 100% wheat straw (control substate); PEWSGM: *Pleurotus eryngii* LGAM 216 in substrate WS:GM (ratio 1:1, w/w) (GM: grape marc); HEBS: *Hericium erinaceus* LGAM 4514 in 100% beech sawdust (BS, control substate); HEOLRP: *Hericium erinaceus* LGAM 4514 in olive pruning residues; CC2WS: *Cyclocybe cylindracea* LGAM 951 in 100% wheat straw (control substate); CC505WS: *Cyclocybe cylindracea* LGAM 961 in 100% wheat straw (control substate); INU2: positive control (inulin, 2% w/v); NC: negative control (basal medium with no carbohydrate source)

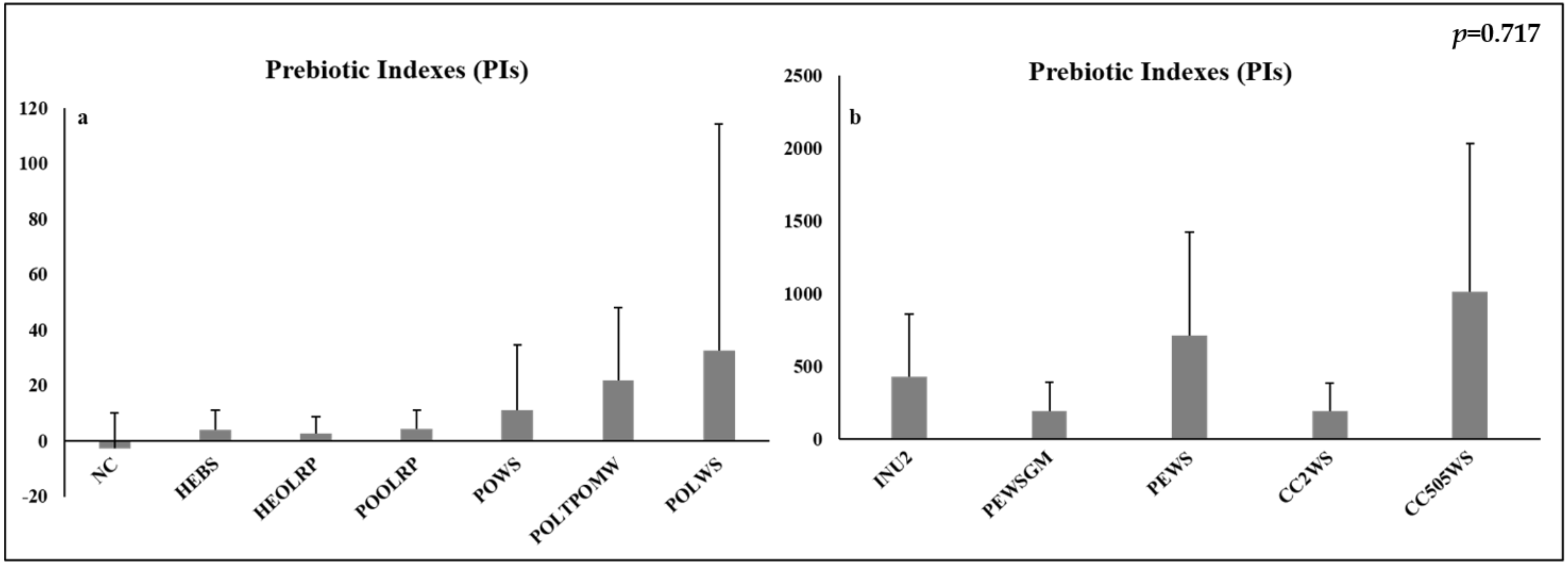

Figure S1a-b: Prebiotic Indexes (PIs) of the tested mushrooms and controls after 24-h fermentation for the first 4 runs of the *in vitro* fermentation experiment. Values are expressed as mean and SD; POWS: *Pleurotus ostreatus* IK 1123 in 100% wheat straw (WS, control substate); POOLRP: *Pleurotus ostreatus* IK 1123 in olive pruning residues (OL); POLWS: *Pleurotus ostreatus* LGM 22 in 100% wheat straw (control substate); POLTPOMW: *Pleurotus ostreatus* LGM 22 in substate OL:TPOMW (ratio 3:1,w/w) (TPOMW: two-phase olive mill wastes); PEWS: *Pleurotus eryngii* LGAM 216 in 100% wheat straw (control substate); PEWSGM: *Pleurotus eryngii* LGAM 216 in substrate WS:GM (ratio 1:1, w/w) (GM: grape marc); HEBS: *Hericium erinaceus* LGAM 4514 in 100% beech sawdust (BS, control substate); HEOLRP: *Hericium erinaceus* LGAM 4514 in olive pruning residues; CC2WS: *Cyclocybe cylindracea* LGAM 951 in 100% wheat straw (control substate); CC505WS: *Cyclocybe cylindracea* LGAM 961 in 100% wheat straw (control substate); INU2: positive control (inulin, 2% w/v); NC: negative control (basal medium with no carbohydrate source).

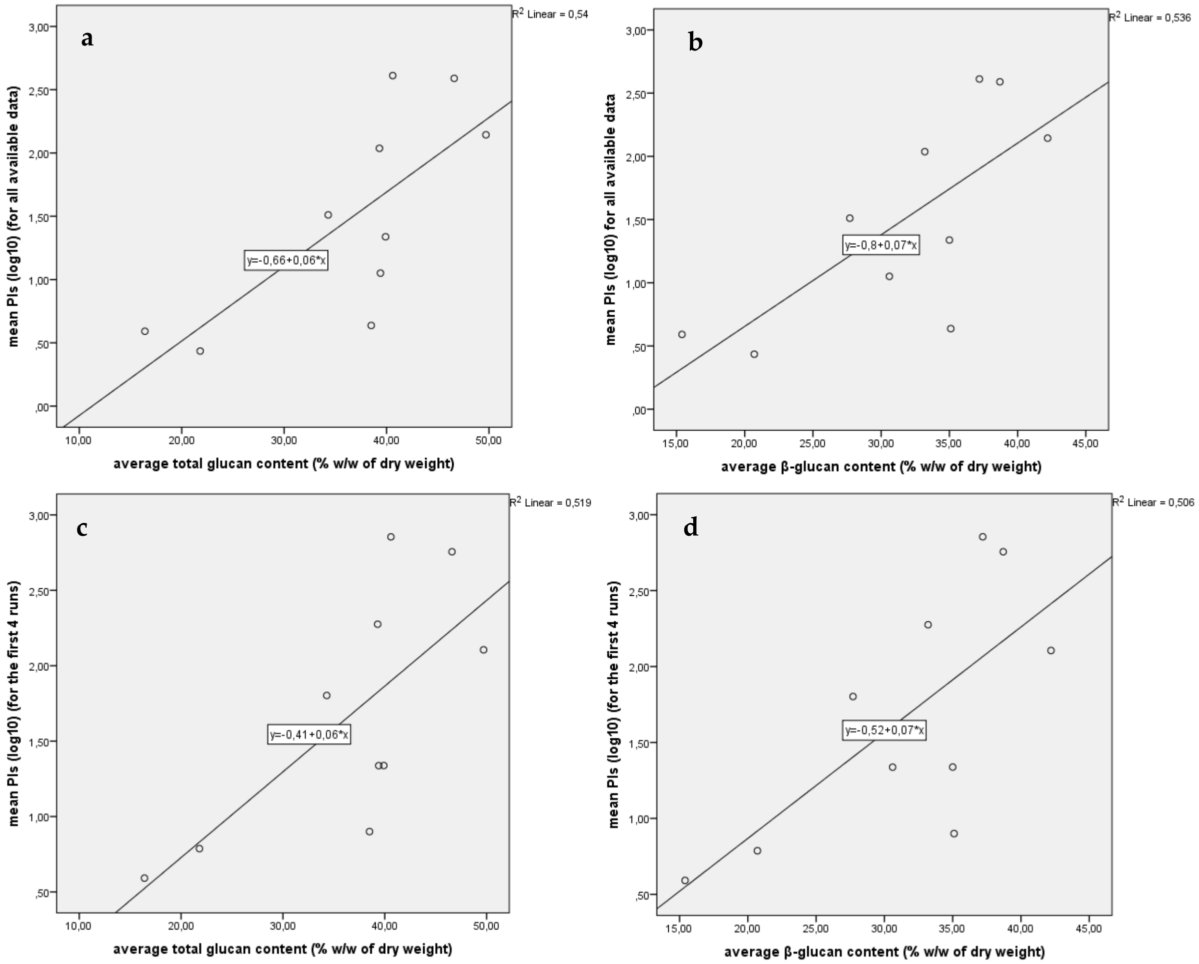

Figure S2: Linear regression analysis of log_{10}-transformed mean Prebiotic Indexes (PIs) of the tested mushrooms with average total glucan content (a,c) and average β-glucan content (b,d) for all available data (a,b) and for the first 4 runs (c,d) of the *in vitro* fermentation experiment.
